# 3-Acetoxy-fatty acid isoprenyl esters from androconia of the ithomiine butterfly *Ithomia salapia*

**DOI:** 10.3762/bjoc.16.228

**Published:** 2020-11-16

**Authors:** Florian Mann, Daiane Szczerbowski, Lisa de Silva, Melanie McClure, Marianne Elias, Stefan Schulz

**Affiliations:** 1 Institute of Organic Chemistry, Technische Universität Braunschweig, Hagenring 30, 38106 Braunschweig, Germany; 2 Institut de Systématique Evolution Biodiversité, Centre National de la Recherche Scientifique, MNHN, Sorbonne Université, EPHE, Université des Antilles, 45 rue Buffon, CP 50, 75005 Paris, France; 3 Laboratoire Écologie, Évolution, Interactions des Systèmes Amazoniens (LEEISA), Université de Guyane, CNRS, IFREMER, 97300 Cayenne, France

**Keywords:** fatty acid esters, mass spectrometry, mimicry, pheromones, pyrrolizidine alkaloids

## Abstract

Male ithomiine butterflies (Nymphalidae: Danainae) have hairpencils on the forewings (i.e., androconia) that disseminate semiochemicals during courtship. While most ithomiines are known to contain derivatives of pyrrolizidine alkaloids, dihydropyrrolizines, or γ-lactones in these androconia, here we report on a new class of fatty acid esters identified in two subspecies, *Ithomia salapia aquinia* and *I. s. derasa.* The major components were identified as isoprenyl (3-methyl-3-butenyl) (*Z*)-3-acetoxy-11-octadecenoate, isoprenyl (*Z*)-3-acetoxy-13-octadecenoate (**12**) and isoprenyl 3-acetoxyoctadecanoate (**11**) by GC/MS and GC/IR analyses, microderivatizations, and synthesis of representative compounds. The absolute configuration of **12** was determined to be *R*. The two subspecies differed not only in the composition of the ester bouquet, but also in the composition of more volatile androconial constituents. While some individuals of *I. s. aquinia* contained ithomiolide A (**3**), a pyrrolizidine alkaloid derived γ-lactone, *I. s. derasa* carried the sesquiterpene α-elemol (**8**) in the androconia. These differences might be important for the reproductive isolation of the two subspecies, in line with previously reported low gene exchange between the two species in regions where they co-occur. Furthermore, the occurrence of positional isomers of unsaturated fatty acid derivatives indicates activity of two different desaturases within these butterflies, Δ9 and Δ11, which has not been reported before in male Lepidoptera.

## Introduction

The Neotropical butterfly tribe Ithomiini (Nymphalidae: Danainae) is very diverse and species-rich, with over 390 species and 50 genera [1–2] and extensively involved in Müllerian mimetic interactions [3]. Ithomiines are well suited for studies on speciation (species formation), as species often consist of multiple subspecies diverging for a number of adaptive traits, such as color pattern or host plants, which can then cause reproductive isolation. As such, they offer an excellent system to study the mechanisms underlying diversification and species recognition. Yet despite growing interests in this tribe, chemical differentiation between taxa has garnered surprisingly little attention until now.

Here we focus on the two closely related taxa, *Ithomia salapia aquinia* and *I. s. derasa.* The two subspecies have somewhat divergent wing color patterns (see Supporting Information File 1, Figure S1) [4], are widely distributed, and parapatric in north-eastern Peru [5]. Despite the geographic overlap in distribution, a recent genetic study showed limited gene flow [4]. Reproductive isolation in mimetic butterflies can be driven by multiple factors, notably non-random mating based on color pattern and/or sexual pheromones [6–8]. Determining whether the closely related subspecies of *I. salapia* differ in the chemical composition of volatiles is, therefore, of great interest.

**Figure 1:**
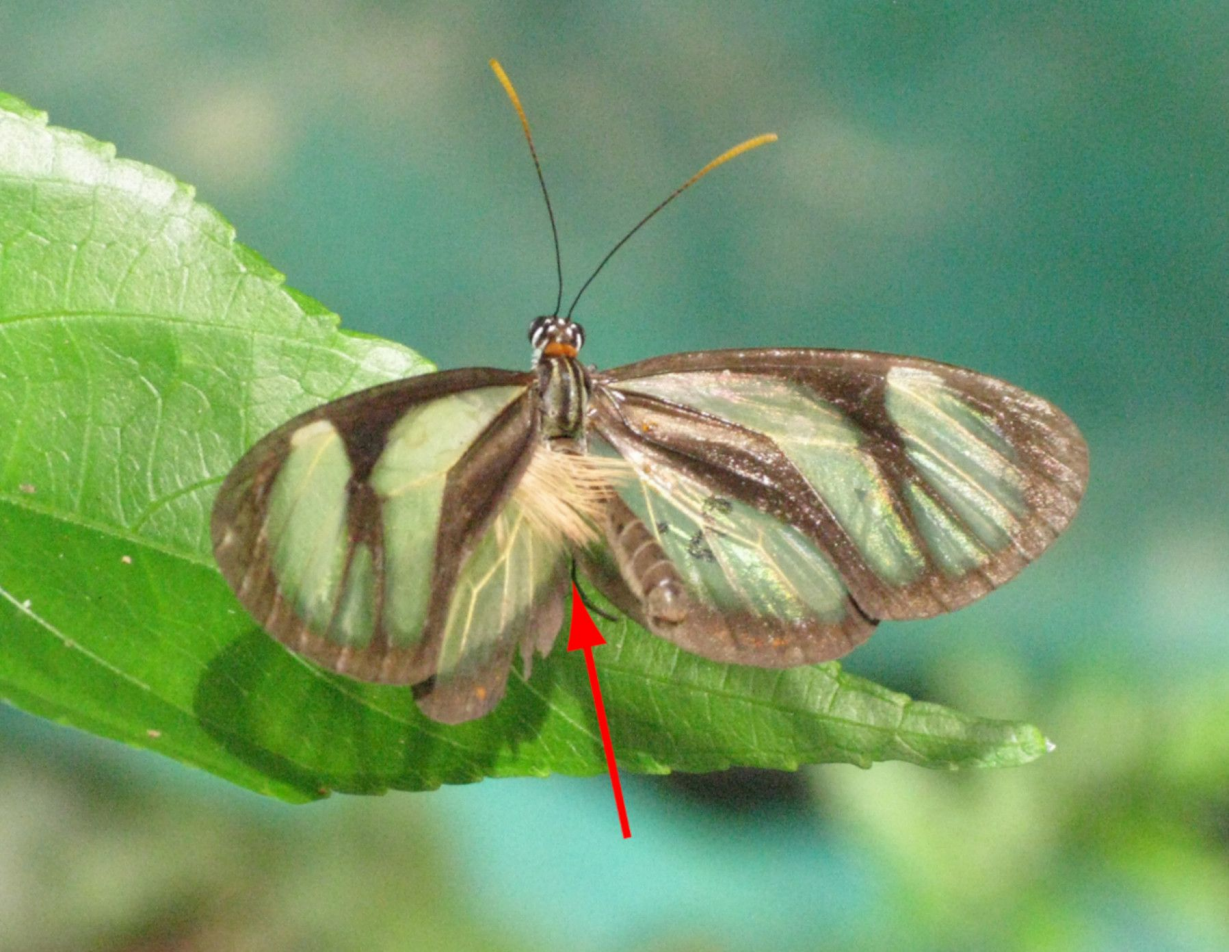
Extended hairs (arrow) of the androconia of a male *Ithomia salapia aquinia* (Photo: Melanie McClure).

**Scheme 1:**
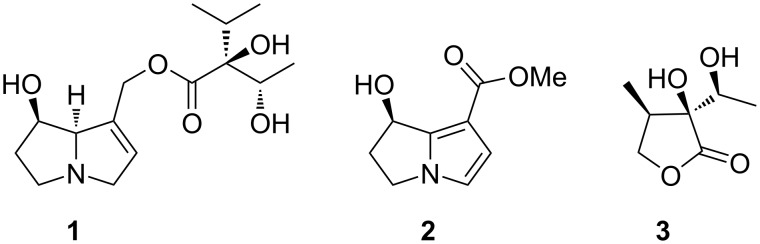
Pyrrolizidine alkaloid lycopsamine (**1**) and the putative pheromone compounds methyl hydroxydanaidoate (**2**) and ithomiolide A (**3**).

All male ithomiine butterflies, including *Ithomia*, possess scent glands on their forewings, so-called androconia, covered with erectable hairpencils (Figure 1). They are used during courtship and are known to contain compounds acting as pheromones for the butterflies [2]. Adult ithomiines sequester pyrrolizidine alkaloids (PAs) pharmacophagously from various plants [9]. These alkaloids are transformed into the alkaloid and pheromone precursor lycopsamine (**1**, Scheme 1) [10–12] that can then be converted either into necine base derived compounds such as methyl hydroxydanaidoate (**2**), or into necic acids derived ones, e.g., ithomiolide A (**3**) [10–12]. While dihydropyrrolizines are also used by other Lepidoptera, e.g., danaines [13–15] or arctiines [16–17], γ-lactones derived from necic acids are specific to ithomiines of more derived taxa [11].

Past studies of the androconia of *Ithomia* have reported the presence of **3** in *Ithomia iphianassa* from Venezuela [10] and in *I. salapia salapia* from Ecuador [11], whereas no PA-derived compounds were found in any *Ithomia* spp. including *I. agnosia agnosia* [11]. Information on non-PA derived compounds in the androconia of ithomiines is mostly lacking, although we recently described (*Z*)-9-hydroxy-6-nonenoic acid and derivatives including dimers and fatty acid conjugates as major constituents of the androconia of *Oleria onega* [18].

Here we report on the chemical composition of the androconia of *Ithomia salapia aquinia* and *I. s. derasa.* A new type of butterfly scent gland constituents, acylated isoprenyl esters of fatty acids, is described, representing a combination of fatty acid and terpene biosynthesis. We also reveal small but reproducible differences between the two subspecies that could potentially be involved in species recognition and reproductive isolation.

## Results

Extracts from the wing androconia of *I. s. derasa* and *I. s. aquinia* were analyzed by GC/MS. The extracts consisted predominately of fatty acid esters with few other compounds (Table 1). While most ithomiines possess PA-derivatives in the androconia [10–12,18–19], only two of the five samples of *I. s. aquinia* contained small amounts of ithomiolide A (**3**), whereas PA derivatives were entirely absent in *I. s. derasa.*

**Table 1:** Compounds found in extracts of the androconia of *Ithomia salapia derasa* and *I. salapia aquinia.* Five individuals of each subspecies were analyzed. Only compounds occurring at least in two individuals of a subspecies are listed. The peak group refers to compounds eluting closely together. The number before the colon indicates the number of individuals carrying this compound, followed by the range of the relative amount.

No	Compound	Peak group	Retention index	*I. salapia derasa*	*I. salapia aquinia*

1	ithomiolide A (**3**)		1219	–	2: 1.91–2.64
2	β-elemene		1388	4: 0.01–0.19	–
3	elemol/hedycaryol isomer		1517	3: 0.02–0.06	–
4	α-elemol (**8**)		1554	5: 0.11–2.88	–
5	elemol/hedycaryol isomer		1662	3: 0.01–0.02	–
6	hexadecenoic acid		1942	–	3: 0.55–5.76
7	hexadecanoic acid		1961	3: 0.02–0.25	3: 0.28–12.88
8	7-heneicosene		2081	3: 0.15–13.97	–
9	heneicosane		2100	3: 0.02–0.54	–
10	octadecenoic acid		2144	4: 0.62–3.69	2: 1.02–7.88
11	isoprenyl 9-hexadecenoate	A	2233	–	3: 0.01–0.33
12	isoprenyl 11-hexadecenoate	A	2244	–	5: 0.32–2.02
13	isoprenyl hexadecanoate	A	2258	–	5: 0.11–2.24
14	tricosane		2300	5: 0.01–0.44	3: 001–0.11
15	11-methyltricosane		2335	4: 0.06–4.04	5: 0.02–0.89
16	eicosenoic acid		2360	3: 0.08–0.96	–
17	isoprenyl octadecadienoate	B	2431	4: 0.01–0.30	–
18	isoprenyl 9-octadecenoate (**10**)	B	2444	5: 0.36–8.27	5: 0.01–12.19
19	isoprenyl 11-octadecenoate	B	2455	2: 0.01–0.02	4: 0.01–0.33
20	isoprenyl octadecanoate	B	2463	5: 0.01–0.32	3: 0.01–0.07
21	isoprenyl 3-acetoxy-11-hexadecenoate	B	2481	5: 0.10–0.40	5: 0.01–0.42
22	isoprenyl 3-acetoxyhexadecanoate	B	2491	5: 0.30–1.32	5: 0.76–4.93
23	pentacosane	B	2500	5: 0.01–0.13	3: 0.01–0.10
24	isoprenyl (2*E*,11Z)-2,11-octadecadienoate	B	2506	4: 0.14–12.63	4: 0.16–0.89
25	isoprenyl (2*E*,13Z)-2,13-octadecadienoate	B	2516	4: 0.01–0.92	4: 0.13–0.38
26	isoprenyl (*E*)-2-octadecenoate	B	2523	5: 0.04–1.96	4: 0.18–0.56
27	11- and 13-methylpentacosane		2535	3: 0.02–0.05	2: 0.01–0.03
28	isoprenyl 3-hydroxy-11-octadecenoate	C	2603	5: 1.10–5.02	–
29	isoprenyl 3-hydroxy-13-octadecenoate (**24**)	C	2622	5: 0.07–0.40	2: 0.03–0.05
30	isoprenyl 3-hydroxyoctadecanoate	C	2626	5: 0.98–2.41	–
31	isoprenyl (*Z*)-3-acetoxy-11-octadecenoate	D	2678	5: 22.72–45.28	5: 14.58–41.42
32	isoprenyl (*Z*)-3-acetoxy-13-octadecenoate (**12**)	D	2692	5: 3.87–14.67	5: 2.38–30.43
33	isoprenyl 3-acetoxyoctadecanoate (**11**)	D	2698	5: 16.01–25.44	5: 26.20–43.73
34	isoprenyl 3-hydroxy-13-eicosenoate		2808	2: 0.01–0.45	–
35	isoprenyl 3-acetoxy-13-eicosenoate	E	2874	5: 4.25–6.81	5: 0.01–1.20
36	isoprenyl 3-acetoxyeicosanoate	E	2891	5: 0.02–0.35	3: 0.01–0.52

**Scheme 2:**
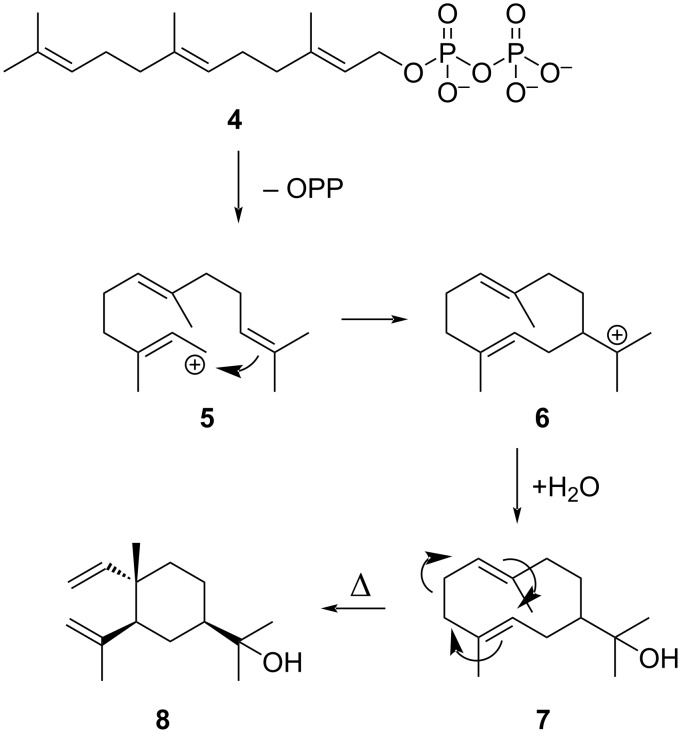
Biosynthetic formation of hedycaryol (**7**) and α-elemol (**8**).

In contrast, the sesquiterpene α-elemol (**8**) was exclusively present in all tested individuals of *I. s. derasa*, together with some related minor sesquiterpenes. This sesquiterpene alcohol is likely formed from hedycaryol (**7**) during GC/MS analysis by a Cope-rearrangement [20–21], indicating that **7** might be originally present in the hairpencils. That said, we cannot disprove that this rearrangement could also occur in the androconia. Hedycaryol is an early product of sesquiterpene biosynthesis, formed by a 1,10-cyclization of the farnesyl cation **5** obtained from farnesyl pyrophosphate (**4**) (Scheme 2). Trapping the cation **6** with water leads to **7**, which in turn might rearrange into **8** [22].

**Figure 2:**
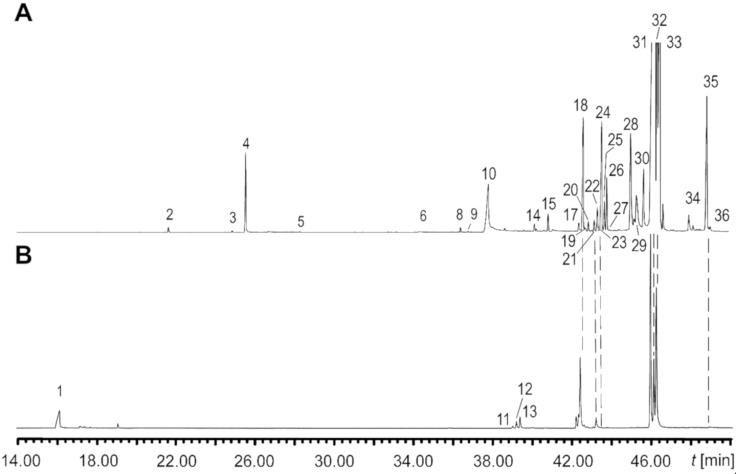
Total ion current chromatogram of androconial extracts of male butterflies of the two subspecies *I. salapia derasa* (A) and *I. s. aquinia* (B). The numbers refer to the entry numbers in Table 1.

**Figure 3:**
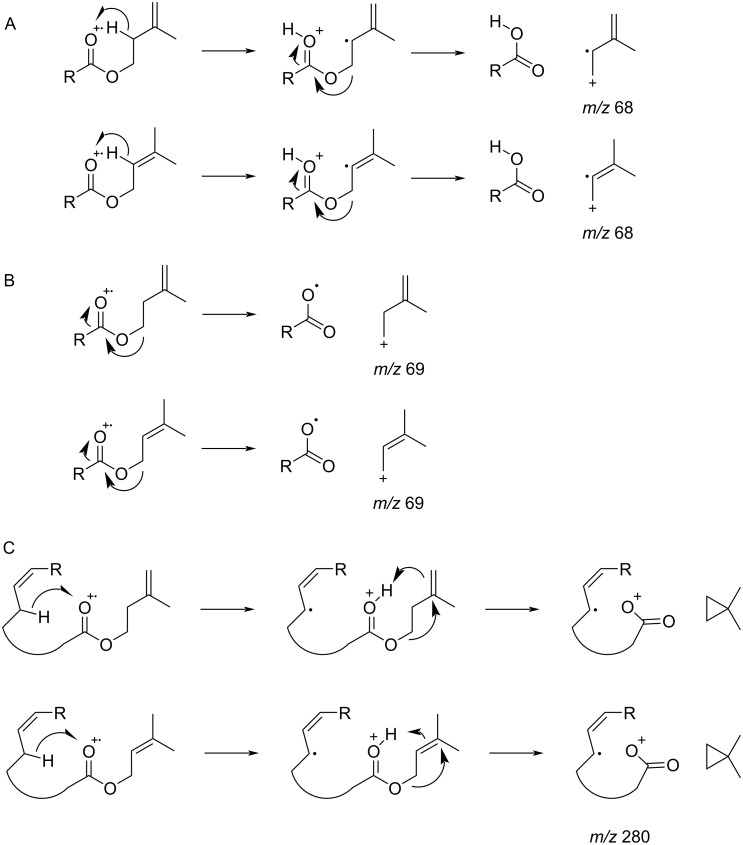
Proposed mass spectrometric formation of characteristic ions in prenyl and isoprenyl esters. Formation of *m*/*z* 68 (A), *m*/*z* 69 (B), and *m*/*z* 280 (C).

**Figure 4:**
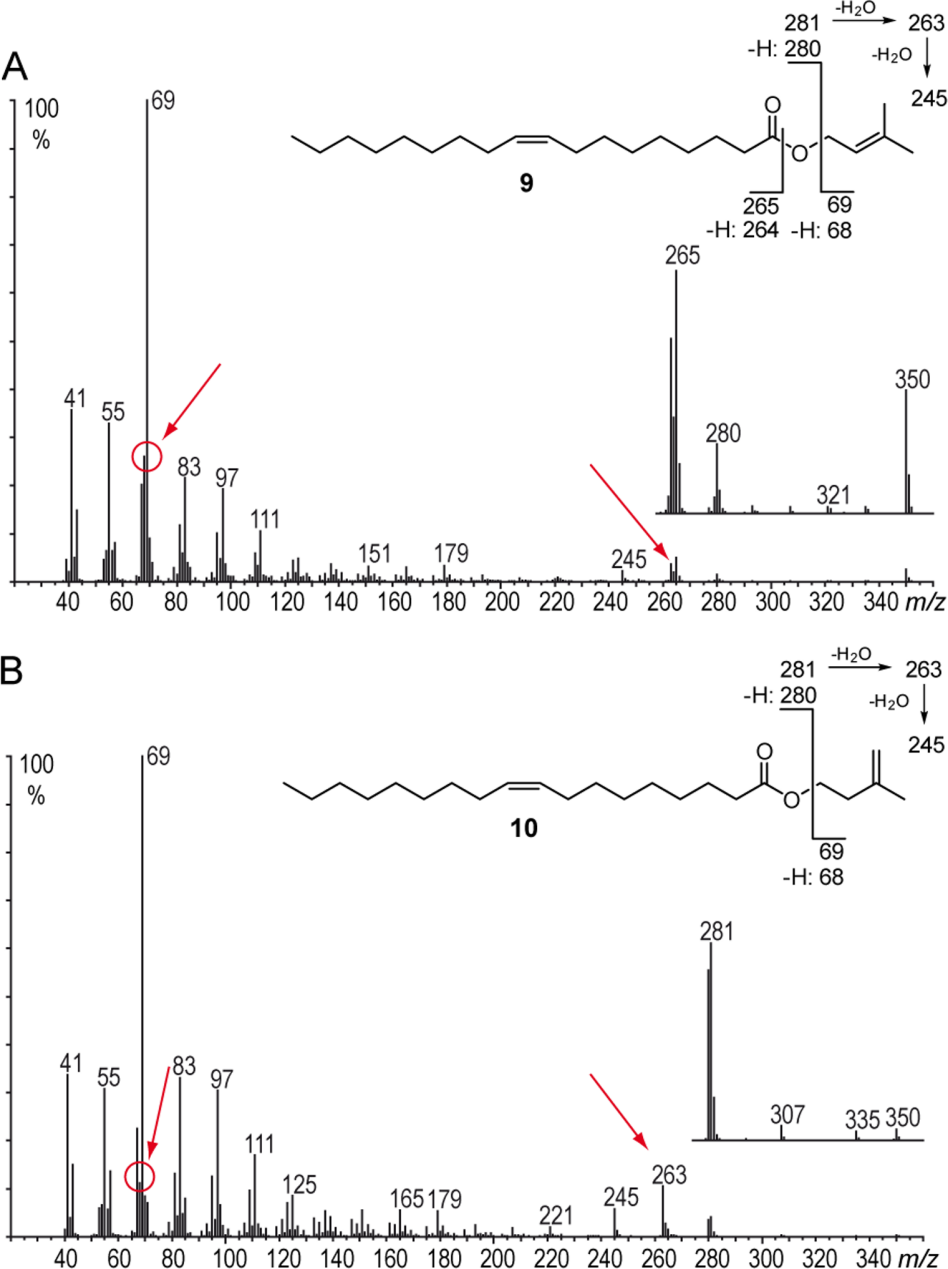
Mass spectra and fragmentation of A: isoprenyl (3-methyl-3-butenyl) 9-octadenoate (**9**) and B: prenyl (3-methyl-2-butenyl) 9-octadecenoate (**10**). Red arrows show characteristics in the mass spectra differentiating prenyl and isoprenyl esters.

The fatty acid ester composition also differed between the two subspecies (Figure 2). Based on their elution order, five groups of compounds were detected, labelled A–E in Table 1. Groups A and B consisted of saturated and unsaturated C_16_ and C_18_ pentenyl esters. These compounds proved to be 3-methyl-3-butenyl esters, which were previously reported in bees [23–24]. Biosynthetically the alcohol part seems to originate from the terpene building block 3-methyl-3-butenyl (isoprenyl) pyrophosphate. Because isoprenyl pyrophosphate is partly converted to 3-methyl-2-butenyl (prenyl) pyrophosphate during terpene biosynthesis, the presence of prenyl esters could not be excluded. Nevertheless, the two ester types can be readily distinguished by EIMS. While 3-methyl-3-butenyl esters of saturated acids have a dominating ion at *m/z* 68, 3-methyl-2-butenyl esters show a peak pair *m/z* 68 and 69 of similar intensity (see Supporting Information File 1, Figure S2), as reported earlier [24]. This difference in the spectra can be explained by the different stabilization of the respective ions (Figure 3). The abundance of *m/z* 68 is higher in isoprenyl esters due to the more stable allyl radical cation (Figure 3A). In contrast, prenyl ester fragmentation produces a stabilized allyl cation *m/z* 69 (Figure 3B), while isoprenyl esters form a less stable homoallyl cation. This situation changes when a double bond is present in the acid part. In both isoprenyl (**9**) and prenyl esters (**10**) ion *m/z* 69 becomes the base peak, but the proportion of *m/z* 68 is higher in the former esters (Figure 4). Other significant differences can be found in the region around the acylium ions. Monounsaturated prenyl esters show the elimination of C_5_H_10_ (M − 70, *m/z* 280 in A), likely formed by rearrangement of an allylic H to the carbonyl group, followed by H-transfer (Figure 3C). Furthermore, the prenyl group can be lost (M − 69, *m/z* 281) and the acylium ion *m/z* 263 is formed. In contrast, isoprenyl esters lack the M − 69 ion, but additionally show acylium +1 and +2 ions (*m/z* 264 and 265 in A).

The location of the double bonds in the unsaturated esters was determined by dimethyl disulfide (DMDS) addition [25–26]. Because the double bond in the isoprenyl side chain would likely interfere, the esters were first transformed into the respective methyl esters via a microreaction with NaOMe [27]. The following DMDS derivatization revealed the presence of two isomers of each chain length, 9- and 11-hexadecenoate, as well as 9- and 11-octadecenoate (Supporting Information File 1, Table S1). Therefore, groups A and B consisted predominately of isoprenyl esters of saturated and unsaturated C_16_- and C_18_-acids.

**Figure 5:**
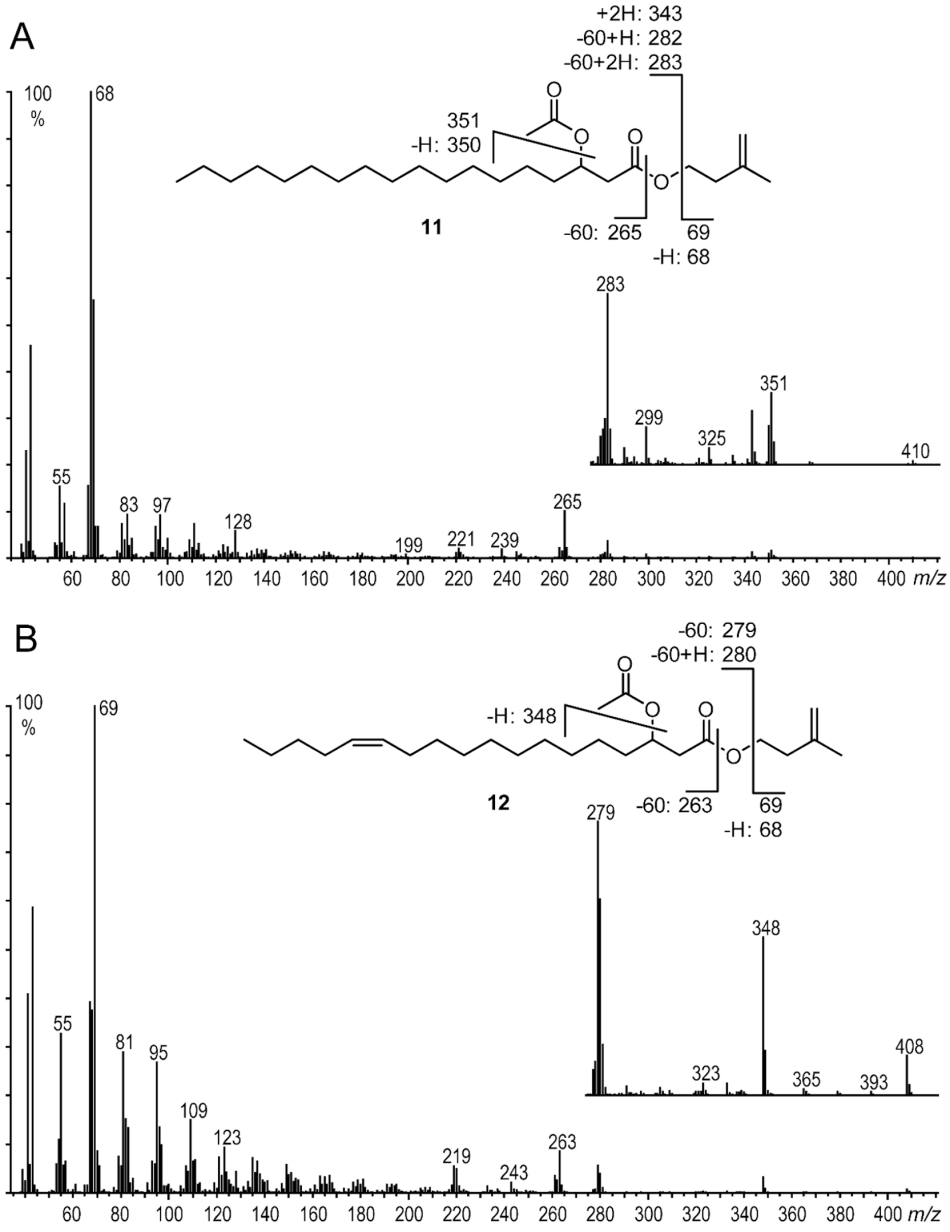
Mass spectra and fragmentation of A: isoprenyl 3-acetoxyoctadecanoate (**11**); B: isoprenyl (*Z*)-3-acetoxy-13-octadecenoate (**12**).

Major components of both subspecies were group D compounds. The peak pair *m/z* 68/69 including the prominent base peak indicated again isoprenyl esters. The mass spectrum of the saturated compound showed a small putative M^+·^ ion at *m/z* 410 and *m/z* 408 for the unsaturated analogs (Figure 5). A loss of 59/60 amu from M^+·^ suggested an acetoxy group located somewhere along the chain. The position could not be derived from the mass spectrum. Nevertheless, the transesterified sample discussed before contained methyl hydroxyalkanoates, which allowed easy location of the hydroxy-group position by GC/MS [28]. The ion *m/z* 103 in the spectra of the three dominating acids confirmed the location of the acetoxy group at C-3 (see Supporting Information File 1, Figure S3). The positions of the double bonds in the methyl esters were determined by DMDS derivatization. The prominent ions present in these adducts allowed the localization of the double bonds in the natural products. Surprisingly, double bonds were found at C-11 and C-13, deducible by the ions *m/z* 145 ([CH_3_SC_7_H_14_]^+^), 261 ([CH_3_CO_2_C_11_H_22_SCH_3_]^+^), 243 (261 − H_2_O), as well as 213 (261 − H_2_O − CH_2_O), and *m/z* 117 ([CH_3_SC_5_H_10_]^+^), 289 ([CH_3_CO_2_C_13_H_26_SCH_3_]^+^), 271 (289 − H_2_O) as well as 241 (289 − H_2_O − CH_2_O), respectively (see Supporting Information File 1, Figure S4). An isomer with a C-9 double bond present in the simple isoprenyl esters was not detected. The configuration of the double bonds was confirmed to be (*Z*) as expected, because GC–DD-IR analyses showed a characteristic C–H stretch band at 3004 cm^−1^ (see Supporting Information File 1, Figure S9) [29–30]. As such, group D consisted of isoprenyl esters of 3-acetoxy-C_18_-fatty acids, a group of compounds not described before in nature. To confirm this, representative isomers were synthesized as outlined below.

**Scheme 3:**
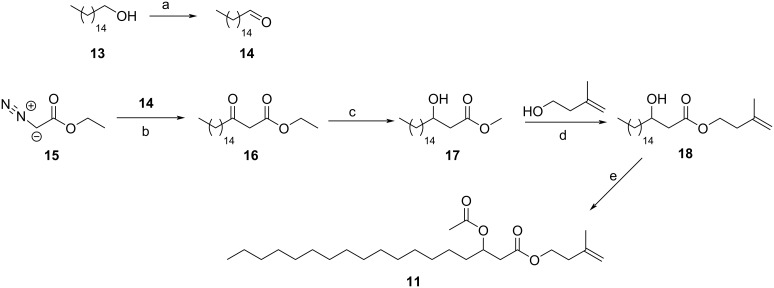
Synthesis of isoprenyl 3-acetoxyoctadecanoate (**11**). a) IBX, EtOAc, 60 °C, 3.15 h, 99%; b) SnCl_2_, CH_2_Cl_2_, rt, 70%; c) NaBH_4_, 12 h, 98%; d) SnOBu_2_, 140°C, 36 h, 78%; e) Ac_2_O, pyridine, DMAP, CH_2_Cl_2_, 12 h rt, 67%.

Isoprenyl 3-acetoxyoctadecanoate (**11**) was synthesized according to Scheme 3. Hexadecanol (**13**) was oxidized to hexadecanal (**14**) using *o*-iodoxybenzoic acid (IBX) [31]. The resulting aldehyde was transformed into β-ketoacid **16** with ethyl diaazoacetate and SnCl_2_ [32], which upon reduction with NaBH_4_ in methanol delivered methyl 3-hydroxyoctadecanoate (**17**). Transesterification was performed with 3-methyl-3-buten-1-ol using distannoxan catalysis [33]. Final acetylation of the hydroxy esters delivered the target compound isoprenyl 3-acetoxyoctadecanoate (**11**). Comparison of mass spectra and retention index confirmed the identity of the naturally occurring compound and **11**.

**Scheme 4:**
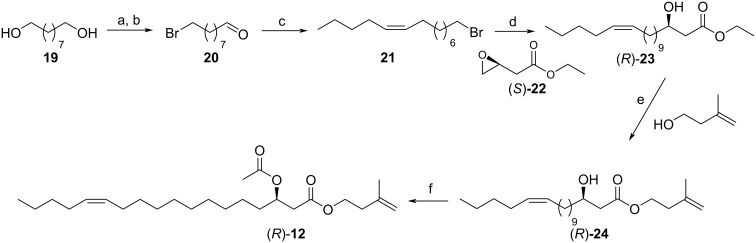
a) 48% HBr_aq_, toluene, 24 h, 110 °C, 79%; b) IBX, EtOAc, 60 °C, 3.15 h, 90%; c) C_5_H_11_PPh_3_Br, LDA, THF, −78 °C, 12 h, 84%; d) i) Mg, **21**, THF, ii) (*S*)-**22**, Cu(I)I, THF, –30 °C, 12 h, 79%; e) SnOBu_2_, 140°C, 36 h, 65%; f) Ac_2_O, pyridine, DMAP, CH_2_Cl_2_, 12 h rt, 74%.

An enantioselective synthesis of isoprenyl (*Z*)-3-acetoxyoctadec-13-enoate (**12**) was performed to verify the structural proposal and to determine the absolute configuration of the natural product (Scheme 4). The commercially available epoxide (*S*)-**22** served as chiral starting material. 1,9-Nonanediol (**19**) was monobrominated and oxidized with IBX to yield 9-bromononanal (**20**). A Wittig reaction with pentylphosphonium bromide resulted in bromoalkene **21** in a 9:1 *Z*/*E*-mixture. In the following step, the Grignard reagent of **21** was converted into the respective Gilman cuprate with Cu(I)I for the selective reaction with the epoxide function of (*S*)-**22** [34]. The hydroxyester **23** was obtained in good yield. The following stannoxane induced transesterification and the final acetylation procedure delivered **12**. The two isomeric natural 3-acetoxyoctadecenyl esters had retention indices of 2678 and 2692, respectively, while synthetic **12** showed an *I* of 2688. Therefore, the second eluting ester is isoprenyl (*Z*)-3-acetoxy-13-octadecenoate, while the earlier eluting one is the 11-isomer.

**Figure 6:**
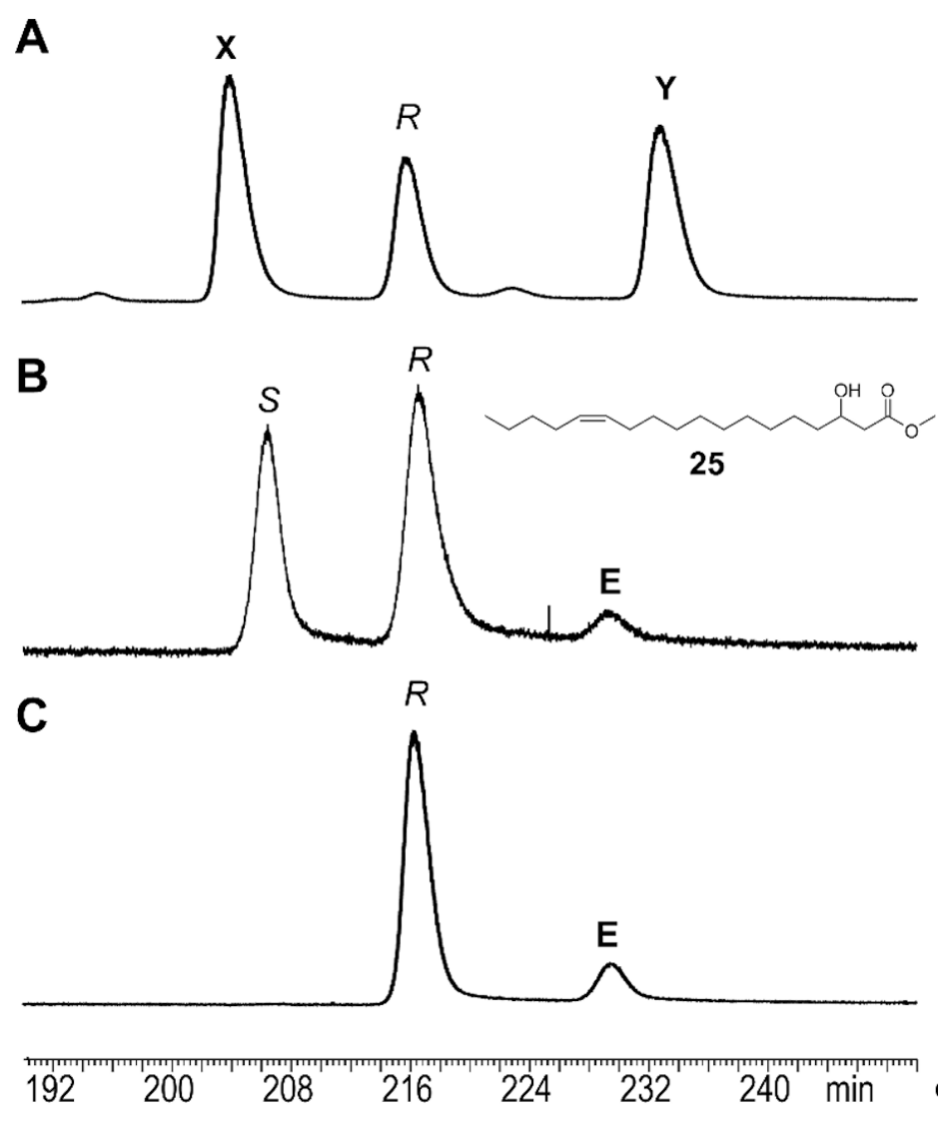
Separation of the enantiomers of methyl (*Z*)-3-hydroxy-13-octadecenoate (**25**) on a β-6-TBDMS hydrodex gas chromatographic phase. A) Natural extract; B) synthetic *rac*-**25**; C) synthetic (*R*)-**25**; **X**: methyl 3-hydroxy-11-octadecenoate; **Y**: methyl 3-hydroxyoctadecanoate; **E**: (*E*)-isomer of (*R*)-**25.** The enantiomer (*S,E*)-**25** elutes together with (*R*,*Z*)-**25**, indicated by the broader base of this peak in B compared to C.

With optically active material in hand, the absolute configuration of **12** was determined by enantioselective gas chromatography. Because direct separation of the large esters seemed to be difficult because of the high elution temperatures needed, we reasoned that the respective methyl 3-hydroxy esters would be much better suited, given the well-known separability of these compounds by chiral GC [35]. Therefore, a natural extract of the androconia and synthetic (*R*)-**12** were transesterified with NaOMe as described above to yield methyl 3-hydroxyoctadecenoates. A synthetic sample of *rac*-**12** obtained from *rac*-**22** was also at hand. The analysis showed that only (*R*)-**12** occurs naturally (Figure 6). Furthermore, the (*Z*)-configuration of the double bond was confirmed, because the minor amount of the (*E*)-isomer, present in the synthetic sample, did not coelute with the natural sample. Although only the configuration of natural **12** was determined to be exclusively (*R*), it seems likely that the other 3-acetoxy esters also show this configuration.

Group E compounds represented bishomologs of **12**, isoprenyl eicosanoate and isoprenyl 13-eicosenoate, determined by DMDS derivatization. Next to these major esters, minor amounts of related esters occurred in some samples. These include deacylated 3-hydroxy esters, isoprenyl 3-hydroxyoctadecenoates and 3-hydroxyoctadecanoate, occurring in group C. Finally, respective elimination products, e.g., isoprenyl 2,11-octadecadienoate and isoprenyl 2-octadecenoate occurred in group B. The location of the C-2 double bond was verified by DMDS derivatization. Because deactivated bonds such as α,β-unsaturated double bonds are too unreactive for DMDS addition, their location can be verified by mass spectrometric fragments missing 2 amu compared to saturated analogs (see Supporting Information File 1, Figure S5) [36]. Hydroxy and α,β-unsaturated esters were not observed during GC of synthetic samples, making an artificial formation during chromatography unlikely.

In addition to the esters, minor amounts of alkanes and alkenes were present, as well as fatty acids. The latter occurred in varying amounts in the samples, maybe depending on variation in quality of the individual sample (see Tables S3 and S4, Supporting Information File 1). Acids are also present in other tissues of the butterflies, unlike the esters. Esters were also not detected in wings of female *I. salapia*.

The major components of the androconia were identical in both subspecies (Table 1). Variations were observed between individuals, and no defined proportion between saturated and unsaturated esters or between different double bond isomers were detected. Nevertheless, differences were present in the minor components and more volatile compounds (Table 1). Sesquiterpene **8** is restricted to *I. s. derasa,* whereas ithomiolide A (**3**) was present exclusively in some of the *I. s. aquinia* samples. C_16_-isoprenyl esters are only found in *I. s. aquinia*, while isoprenyl 3-acetoxy-13-eicosenoate, abundant in *I. s. derasa*, occurs only in trace amounts in *I. s. aquinia.*

## Discussion

The occasional occurrence of PA derivative **3** in two samples of *I. s. aquinia* may depend on the availability of the PA precursor in the wild. It might be that all individuals devoid of **3** simply had no access to PAs and/or that its absence is a specific trait of *I. s*. *derasa*. In contrast, elemol/hedycaryol (**8**) is specific to the latter subspecies*.* Although sesquiterpenes are common in plants, the occurrence of a single sesquiterpene might indicate individual biosynthesis in this subspecies or specific take-up, because plant sesquiterpenes usually occur in mixtures. Furthermore, hedycaryol is a quite simple sesquiterpene, needing only one biosynthetic cyclization step from the universal sesquiterpene precursor farnesyl pyrophosphate (Scheme 2) [22]. The differences in the isoprenyl esters reported are present in all individuals tested, pointing to distinct differences in activity of biosynthetic enzymes between the two subspecies.

Fatty acid esters, which were repeatedly reported to occur in androconia and male scent glands of butterflies [37–41], have been proposed to function e.g., as fixatives for more volatile pheromones [37], but their exact function remains mostly unknown. Because of the quite low volatility of the isoprenyl esters, especially of the major acetoxy esters, olfactory activity seems likely only in close vicinity of the male wings, although the evaporation rate might be increased by erection of the androconia hairpencils (Figure 1). Alternatively, direct or close contact might be needed for detection, probably taking place during contact of the female antennae with the male wings. What this potential signal might indicate remains speculative.

**Scheme 5:**
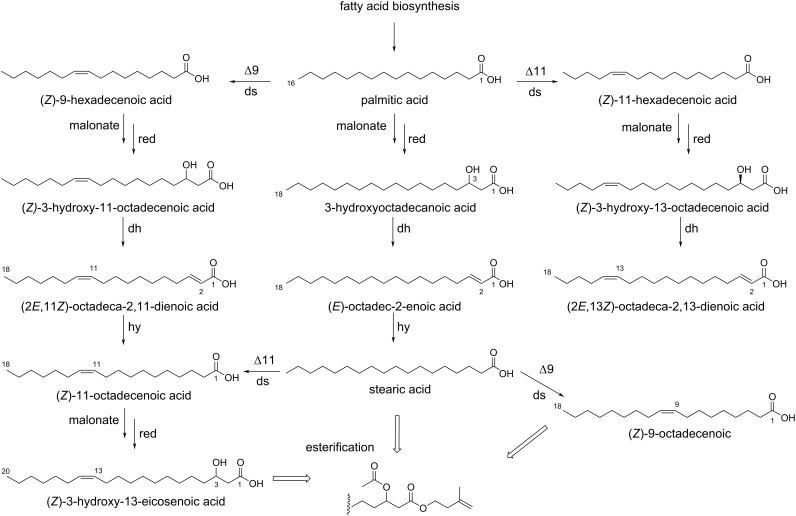
Proposed biosynthetic pathway of fatty acids leading to the observed regioisomers of the isoprenyl esters**.** All acids shown were found in form of their isoprenyl esters. (*Z*)-9-Hexadecenoic acid is obtained from palmitic acid by a Δ9-desaturase (ds). Malonate elongation and reduction (red) leads to (*Z*)-3-hydroxy-11-octadecenoic acid, an intermediate of the fatty acid elongation cycle. The following elimination by a dehydratase (dh) leads to (2*E*,11*Z*)-2,11-octadecadienoic acid and after hydrogenation (hy) to (*Z*)-11-octadecenoic acid, completing the C_2_-elongation. A second elongation furnishes (*Z*)-3-hydroxy-13-eicosenoic acid. Similarly, a Δ11-desaturase gives (*Z*)-11-hexadecenoic acid, (*Z*)-3-hydroxy-13-octadecenoic acid and (2*E*,13*Z*)-2,13-octadecadienoic acid. Both desaturases might also act on octadecanoic acid, but only the elongation of (*Z*)-11-octadecenoic acid can be observed, leading to (*Z*)-3-hydroxy-13-eicosenoic acid. The saturated 3-hydroxyoctadecanoic and stearic acids as well as (*E*)-2-octadecenoic acids are obtained similarly directly from palmitic acid. The proposed biosynthesis likely takes place in form of the conjugated acids, e.g., coenzyme A esters or acyl carrier proteins. Finally, the acids are converted into the isoprenyl esters and the hydroxy acids are acylated.

Nevertheless, the unusual location of the double bonds suggests an active function of the isoprenyl ester as signaling compounds [42]. Unsaturated fatty acid derivatives in pheromone glands are typically introduced by desaturases acting on saturated precursors. In the biosynthetically well-known butterfly genus, *Bicyclus*, a Δ11-desaturase, also often involved in moth pheromone biosynthesis, leads to derivatives of Δ11-C_16_ and C_18_-acids [43]. The fatty acid derivatives present in the male hairpencils of the danaine butterfly *Lycorea ceres ceres* also indicate the presence of a Δ11-desaturase [44]. In contrast, products consistent with Δ9-desaturase activity are present in androconia of the genus *Heliconius* [45]. Unlike these species, *Ithomia* seems to use at least two different desaturases, Δ9 and Δ11, leading to regioisomeric mixtures of isoprenyl esters. The position of the double bonds in the acyl chain of the esters can be explained by a biosynthetic pathway described in detail in Scheme 5. The double bond distribution is consistent with both desaturases acting on palmitic acid, leading to the respective hexadecenoic acids. These acids are the starting material for an additional elongation cycle of the fatty acid biosynthesis, leading *en route* to 3-hydroxy- and 2-alkenoic acids and finally to 11- and 13-octadecenoic acids. While the latter free acid was not observed, 9-octadecenoic acid was also present, formed likely by action of the Δ9-desaturase on stearic acid.

3-Acetoxylated fatty acid esters are rarely found as natural products. Ethyl (*S*)-3-acetoxyeicosanoate and longer analogs are produced by the plant *Schizolaena hystrix* [46], but similar compounds from insects are unknown. Related are cactoblastins used as trail-following pheromones by *Cactoblastis cactorum* [47], which represent methyl esters of 3-hydroxy fatty acids acylated at O-3 with another fatty acid (structures see Supporting Information File 1, Figure S8).

The isoprenyl fatty acid esters are not restricted to the genus *Ithomia* within the Ithomiini. Preliminary analysis also revealed that these esters are also constituents of the androconia of e.g. *Hypothyris anastasia*, *Hyposcada illinissa*, *H. anchiala*, or *Melinaea menophilus*. In contrast, 9-hydroxynonanoic acid derived acids and esters are currently only reported from *Oleria* [18].

## Conclusion

In summary, we here describe a group of esters, never before reported in nature, 3-acetoxyacyl isoprenyl esters from *Ithomia salapia*. The large amounts of these esters in the androconia and the specialized enzymes needed to produce them seem to indicate a pheromonal function of them, especially at close range. Differences in composition between the two subspecies suggest a possible role of the chemical bouquet in reproductive isolation, although other factors, such as wing color pattern, can also act as a reproductive barrier.

## Supporting Information

File 1 Butterfly photos, mass, IR and NMR spectra, experimental procedures and analysis of individuals.
