# Supplementary material for: 3-Acetoxy-fatty acid isoprenyl esters from androconia of the ithomiine butterfly Ithomia salapia
Source: Beilstein J Org Chem. 2020 Nov 16;16:2776–87. doi: 10.3762/bjoc.16.228 (PMC7684689; doi:10.3762/bjoc.16.228)
Supplement: File 1 — Butterfly photos, mass, IR and NMR spectra, experimental procedures and analysis of individuals. [file Beilstein_J_Org_Chem-16-2776-s001.pdf]

BEILSTEIN JOURNAL OF ORGANIC CHEMISTRY

Supporting Information

for

3-Acetoxy-fatty acid isoprenyl esters from androconia of the ithomiine butterfly *Ithomia salapia*

Florian Mann, Daiane Szczerbowski, Lisa de Silva, Melanie McClure, Marianne Elias and Stefan Schulz

Beilstein J. Org. Chem. **2020,** *16,* 2776–2787. doi:10.3762/bjoc.16.228

Butterfly photos, mass, IR and NMR spectra, experimental procedures and analysis of individuals

License and Terms: This is a supporting information file under the terms of the Creative Commons Attribution License (https://creativecommons.org/licenses/by/4.0). Please note that the reuse, redistribution and reproduction in particular requires that the authors and source are credited.
The license is subject to the *Beilstein Journal of Organic Chemistry* terms and conditions: (https://www.beilstein-journals.org/bjoc)

Contents

1. Photos	S2
2. Mass Spectrometry	S3
3. Experimental part	S10
4. IR spectra of natural compounds	S22
5. NMR spectra	S23
6. References	S31

1. Photos

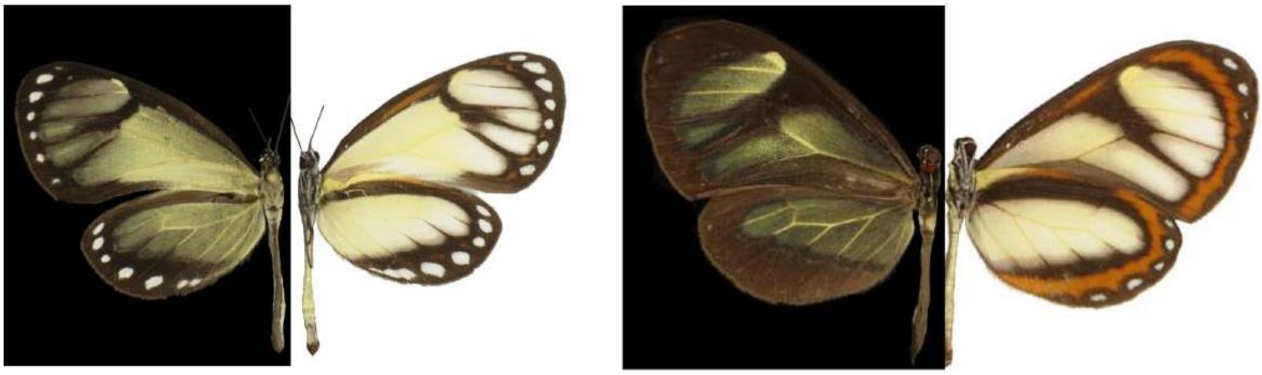

Figure S1. ***Ithomia salapia derasa*** **(left) and** ***I. s. aquinia*** **(right).** The left side of the butterflies shows the dorsal sides against a dark background to highlight transparency, the right shows the ventral sides. Photos by Céline Houssin [S1].

2. Mass spectrometry

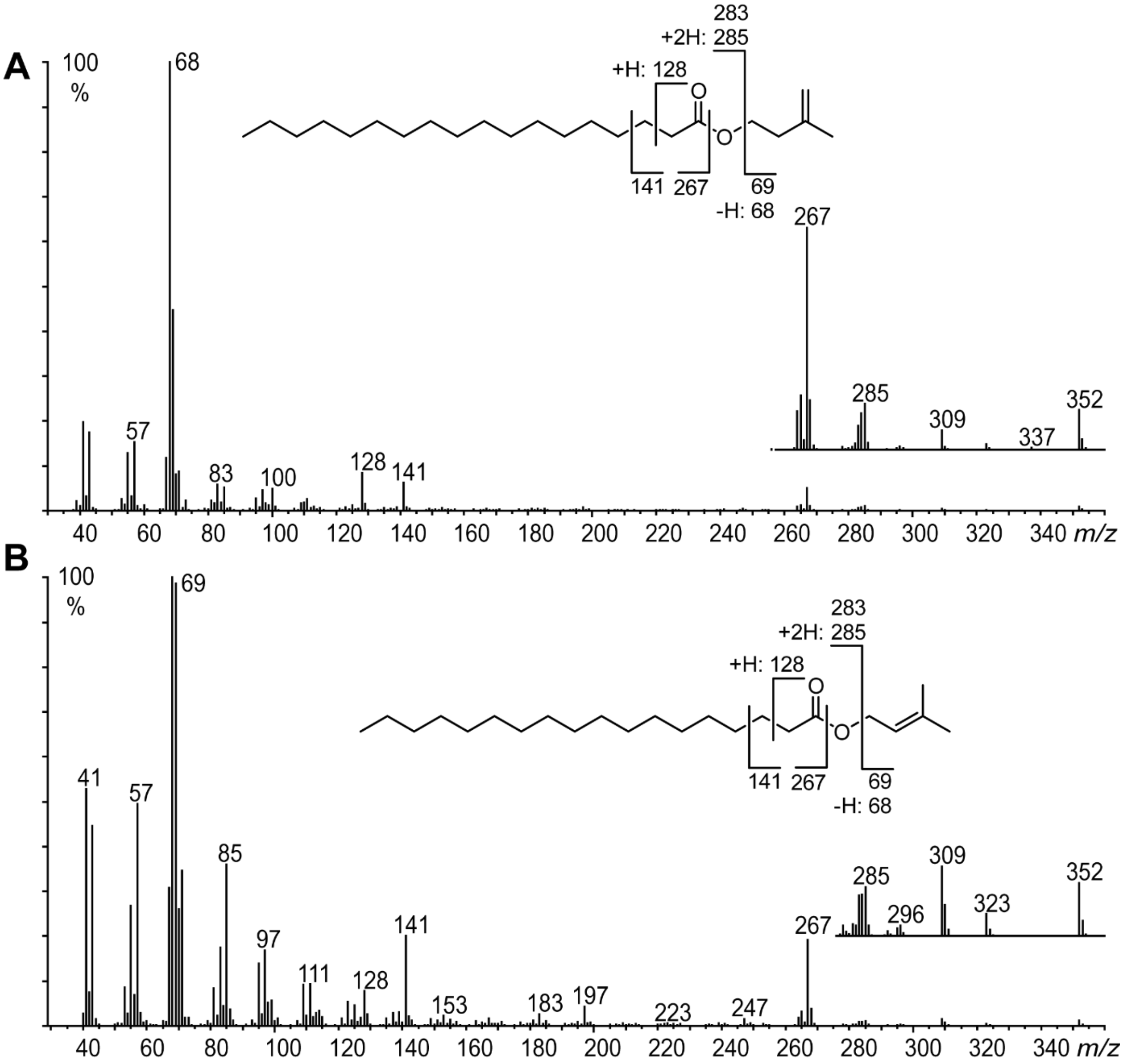

Figure S2. Mass spectra and mass spectrometric fragmentation of 3-methyl-3-butenyl octadecanoate (A) and 3-methyl-2-butenyl octadecanoate (B).

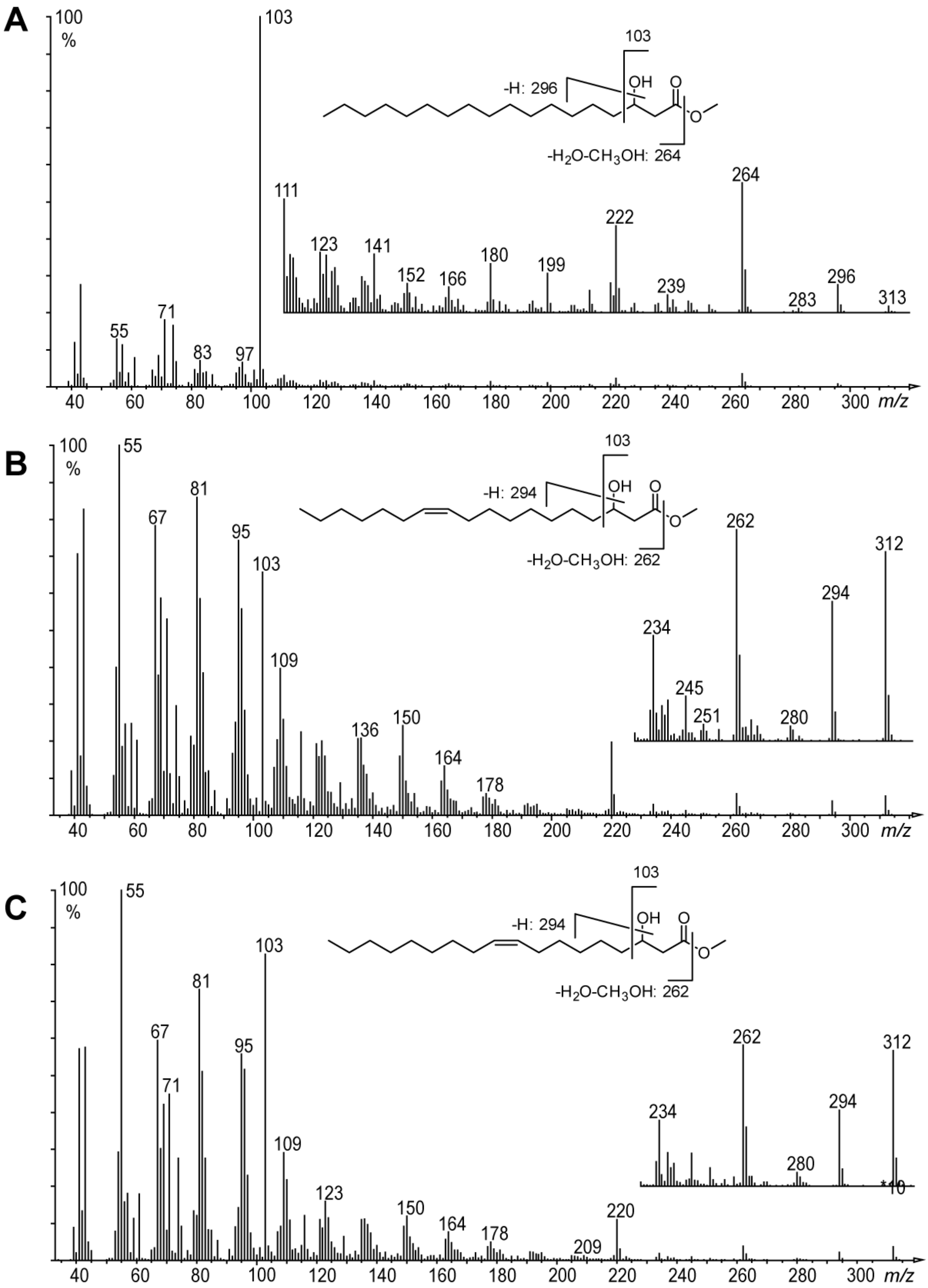

Figure S3. Mass spectra and mass spectrometric fragmentation of methyl 3-hydroxyoctadecanoate (A) and methyl 3-hydroxyoctadecenoates (B, C). Assignment of the double bond position is not possible from the spectra. Nevertheless, the later eluting compound usually has the double bond closer to the ω-end on apolar gas chromatographic phases, indicating that B likely represents methyl (*Z*)-3-hydroxy-11-octadecenoate, while C

might be methyl (*Z*)-3-hydroxy-13-octadecenoate. The more abundant ion *m/z* 103 in C also seems to indicate a larger distance between double-bond and OH-group compared to B, leading to less interaction between the two preferentially ionized π-bonds in the compounds.

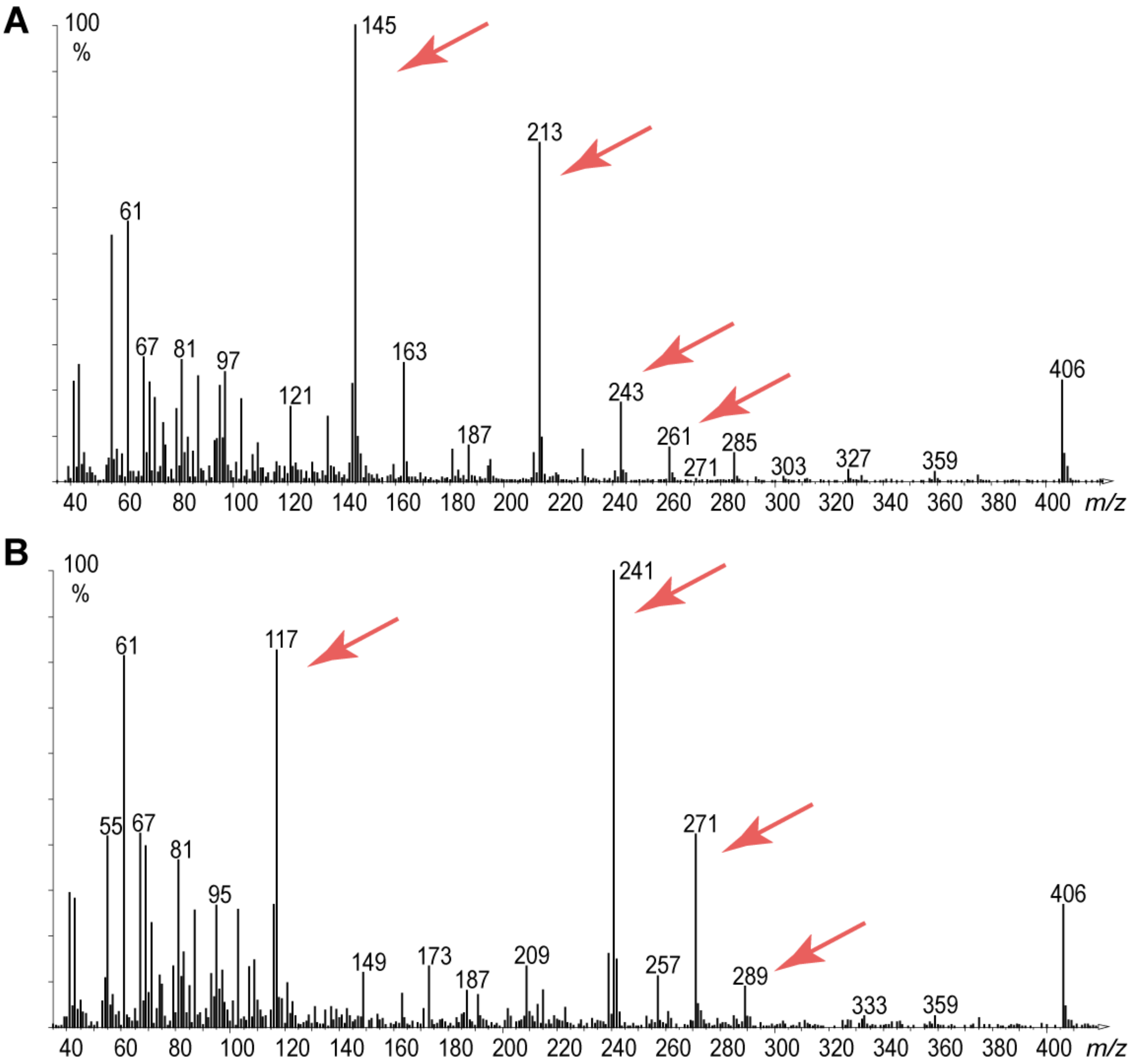

Figure S4. Mass spectra of DMDS adducts of methyl 3-hydroxy-11-octadecenoate (A) and of methyl 3-hydroxy-13-octadecenoate (B). Red arrows indicate characteristic ions used for localization of the double bond. See main text for explanation.

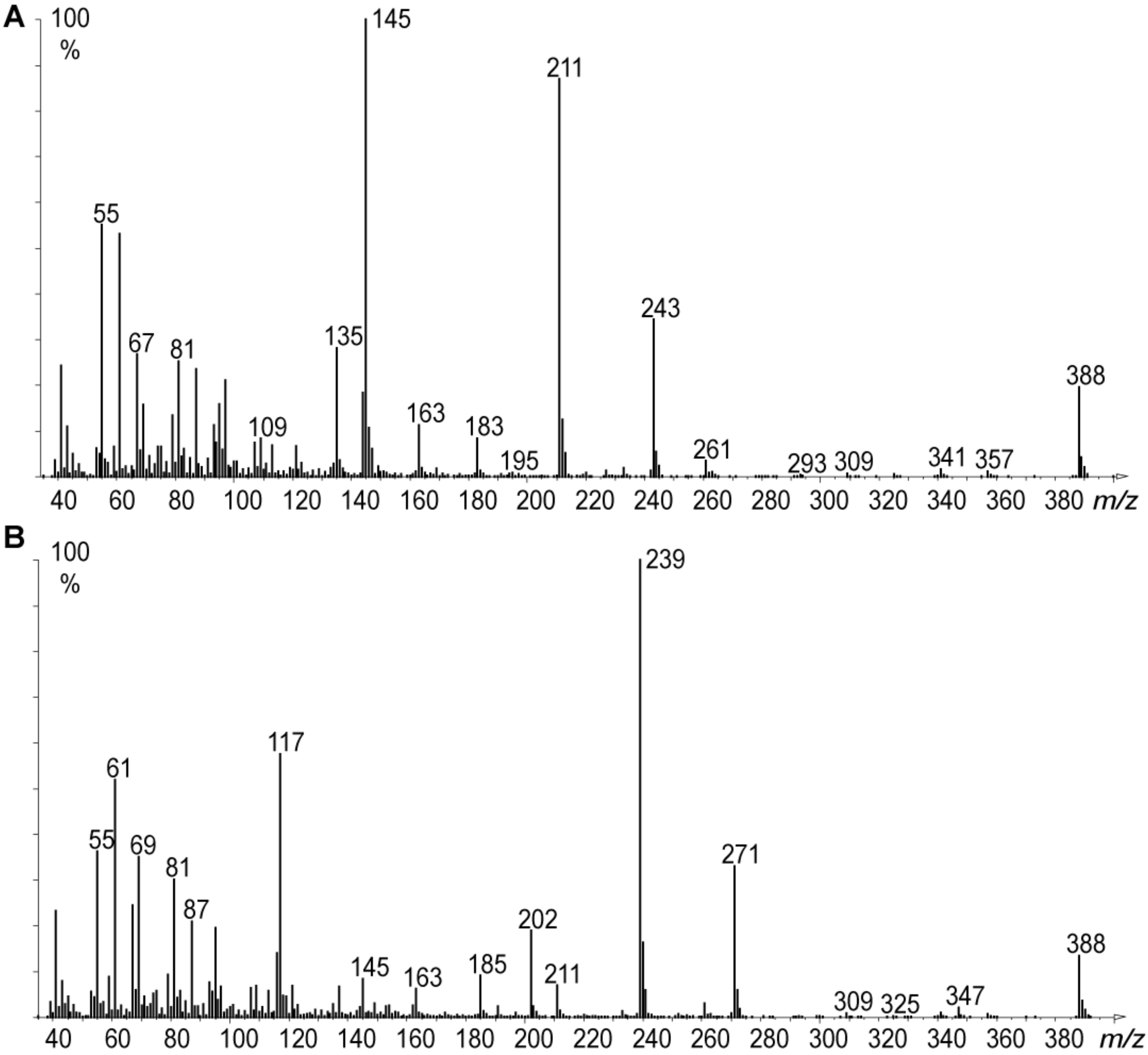

Figure S5. Mass spectra of DMDS adducts of methyl 2,11-octadecadienoate (A) and methyl 2,13-octadecadienoate (B). Spectrum A is similar to that of methyl 11-octadecenoate (Table S1), but shows 2 amu lower ions, *m/z* 211, 243 and 388. This indicates a double bond at C-2, because this position is the only one that does not react with DMDS due to its lower reactivity because of the electron-withdrawing conjugated ester group.

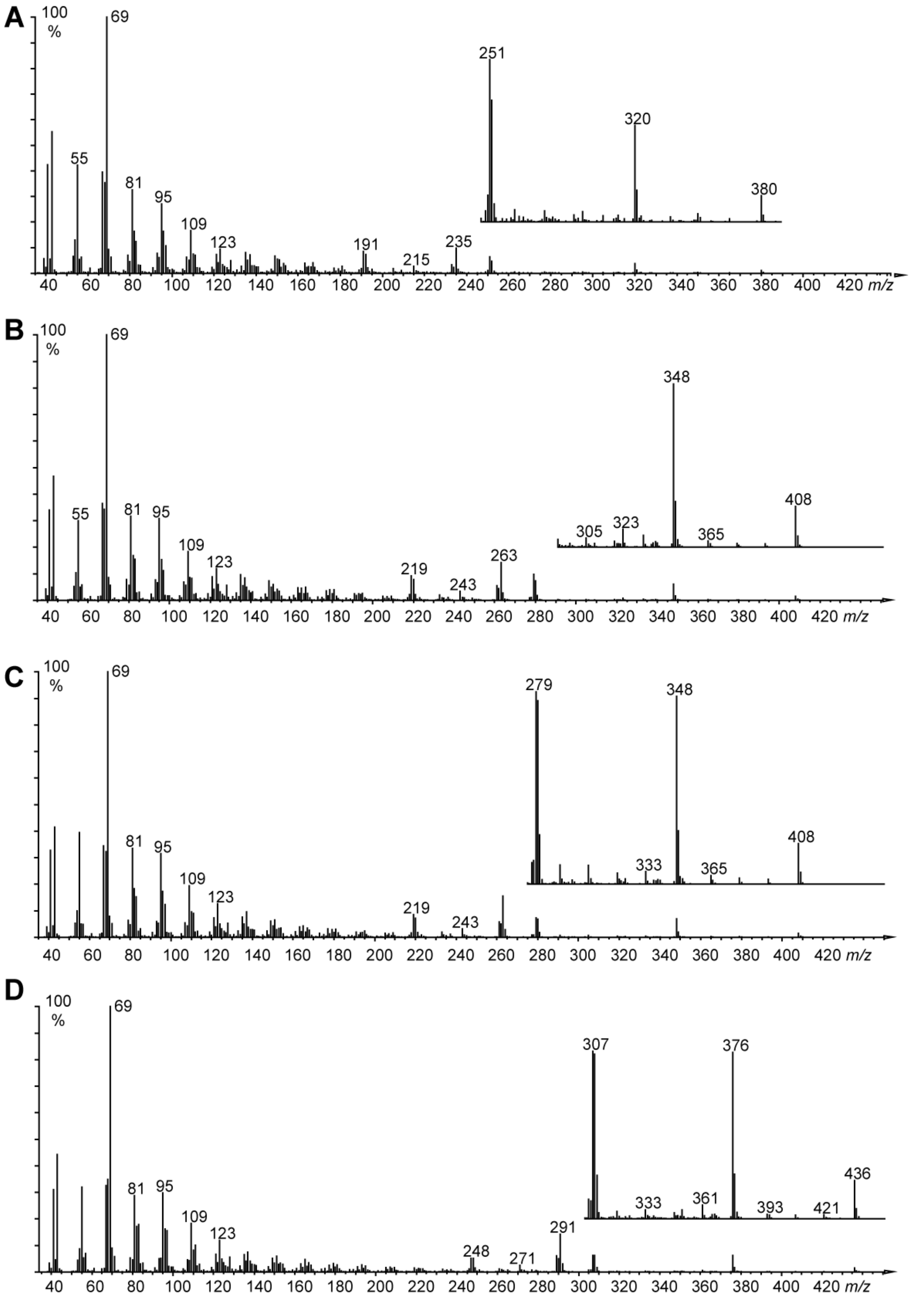

Figure S6. Mass spectra of isoprenyl 3-acetoxy-11-hexadecenoate (A), isoprenyl 3-acetoxy-11-octadecenoate (B), isoprenyl 3-acetoxy-13-octadecenoate (C), and isoprenyl 3-acetoxyeicosenoate (D) from extracts of androconia of *Ithomia salapia*.

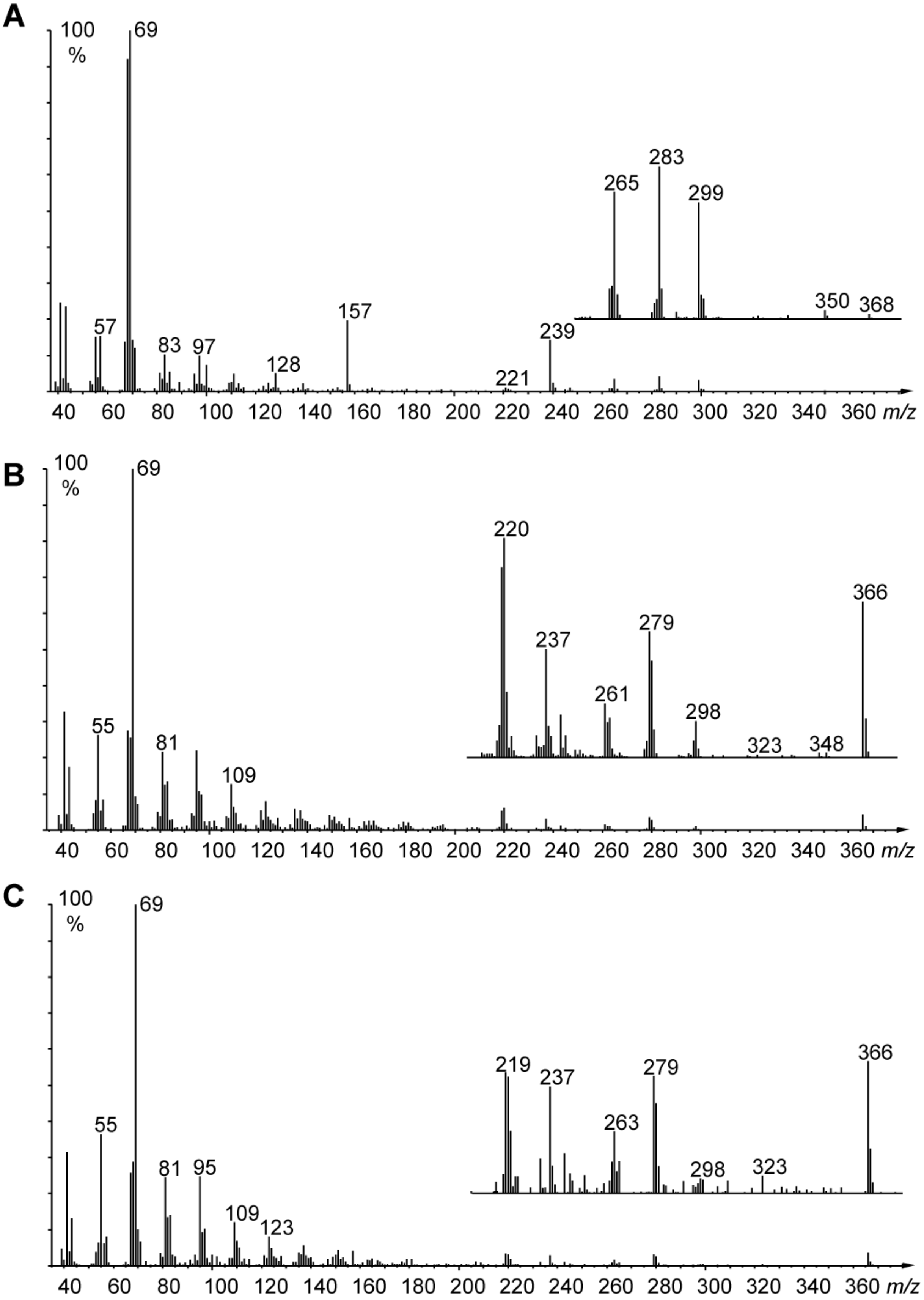

Figure S7. Mass spectra of isoprenyl 3-hydroxyoctadecanoate (A), isoprenyl 3-hydroxy-11-octadecenoate (B), and isoprenyl 3-hydroxy-13-octadecenoate (C) from extracts of androconia of *Ithomia salapia*.

Table S1. Localization of double-bonds in DMDS-adducts of methyl esters obtained by transesterification of isoprenyl esters. Characteristic ions.

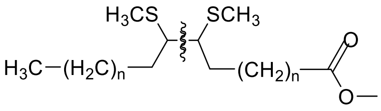

	$[CH_3S(CH_2)_nCH_3]^+$	$[CH_3S(CH_2)_nCOOCH_3]^+$	$[CH_3S(CH_2)_nCOOCH_3\text{-}CH_3OH]^+$	M^+
Methyl (*Z*)-9-hexadecenoate	145	217	185	362
Methyl (*Z*)-11-hexadecenoate	117	245	213	362
Methyl (*Z*)-9-octadecenoate	173	217	185	390
Methyl (*Z*)-11-octadecenoate	145	245	213	390
Methyl (*Z*)-13-eicosenoate	145	273	241	418

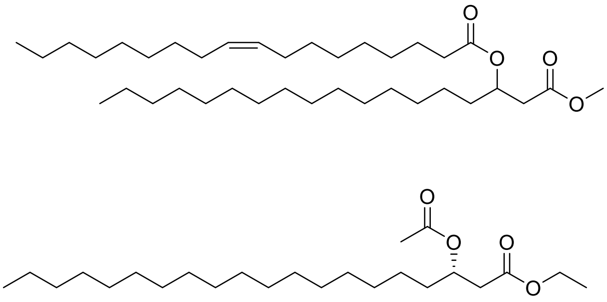

Figure S8. Related natural products: Representative structure of cactoblastins (top) and ethyl (*S*)-3-acetoxyeicosanoate (bottom).

3. Experimental part

3.1 Samples

Male butterflies of *Ithomia salapia aquinia* and *I. s. derasa* were collected in north-eastern Peru from 2011-2012 (sampling information in Table S2 below). The androconial hairpencils were dissected and extracted in ultrapure dichloromethane shortly after capture. Samples were kept at -20ºC until analysis.

Table S2. Sampling information including date and location of collection, GPS positions and altitude.

Sample	Sub-species	Date of collection	Location	GPS	Altitude
LdeS11-34	*I. s. aquinia*	30 January 2011	Km5 Shapaja - Chazuta	6°35'424"S ; 76°13'394"W	200m
LdeS11-81	*I. s. derasa*	2 February 2011	Puente Serranoyacu	5°40'316"S ; 77°40'287"W	1200m
LdeS11-82	*I. s.derasa*	2 February 2011	Puente Serranoyacu	5°40'316"S ; 77°40'287"W	1200m
LdeS11-270	*I. s.derasa*	9 February 2011	Km41.5 Tarapoto - Yurimaguas	6°24'308"S ; 76°15'903"W	400m
LdeS11-272	*I. s. aquinia*	9 February 2011	Km41.5 Tarapoto - Yurimaguas	6°24'308"S ; 76°15'903"W	400m
LdeS11-273	*I. s. aquinia*	9 February 2011	Km41.5 Tarapoto - Yurimaguas	6°24'308"S ; 76°15'903"W	400m
LdeS11-455	*I. s. aquinia*	10 March 2011	Km24 Yurimaguas - Tarapoto (Km6 - Micaela Bastidas)	5°56'660"S ; 76°14'669"W	180m
LdeS11-1293	*I. s.derasa*	26 November 2011	Puente Aguas Verdes	5°41'077"S ; 77°39'487"W	1100m
LdeS11-1294	*I. s.derasa*	26 November 2011	Puente Aguas Verdes	5°41'077"S ; 77°39'487"W	1100m
LdeS11-1758	*I. s. aquinia*	21 January 2012	Km24 Yurimaguas - Tarapoto (Km6 - Micaela Bastidas)	5°56'660"S ; 76°14'669"W	180m

Table S3. Relative proportions of compounds occurring at least two times in five samples of androconia of male *I. s. aquinia.* Column heads show internal sample code number and sample collection number (see Table S2).

	ISA-1 LDES11-273	ISA-2 LDES11-455	ISA-3 LDeS11-1758	ISA-5 LdeS-11-34	ISA-6 LdeS11-472
Ithomiolide A (**3**)	2.64	0.00	0.00	0.00	1.91
Hexadecenoic acid	5.76	0.00	2.32	0.55	0.00
Hexadecanoic acid	12.88	0.00	2.38	0.28	0.00
Octadecenoic acid	7.88	0.00	1.02	0.00	0.00
Isoprenyl 9-hexadecenoate	0.01	0.33	0.01	0.00	0.00
Isoprenyl 11-hexadecenoate	0.32	1.28	0.51	1.92	2.02
Isoprenol hexadecanoate	0.11	2.24	0.32	1.30	0.82
Tricosane	0.11	0.04	0.01	0.00	0.00
11-Methyltricosane	0.89	0.02	0.11	0.02	0.10
Isoprenyl octadecadienoate	0.00	2.23	0.01	0.00	0.00
Isoprenyl 9-octadecenoate	0.01	12.19	0.67	0.67	0.71
Isoprenyl 11-octadecenoate	0.33	0.29	0.01	0.01	0.00
Isoprenyl octadecanoate	0.00	0.07	0.02	0.01	0.00
Isoprenyl 3-acetoxy-11-hexadecenoate	0.42	0.07	0.34	0.03	0.01
Isoprenyl 3-acetoxyhexadecanoate	4.93	1.98	2.33	0.85	0.76
Pentacosane	0.10	0.02	0.01	0.00	0.00
Isoprenyl (2*E*,11*Z*)-2,11-octadecadienoate	0.44	0.00	0.16	0.89	0.49
Isoprenyl (2*E*,11*Z*)-2,13-octadecadienoate	0.30	0.00	0.13	0.30	0.38
Isoprenyl (*E*)-2-octadecenoate	0.48	0.00	0.18	0.56	0.35
11-and 13-Methylpentacosane	0.03	0.00	0.01	0.00	0.00
Isoprenyl 3-hydroxy-13-octadecenoate (**24**)	0.00	0.00	0.03	0.05	0.00
Isoprenyl (*Z*)-3-acetoxy-11-octadecenoate	14.58	41.42	35.50	40.69	35.83
Isoprenyl (*Z*)-3-acetoxy-13-octadecenoate (**12**)	2.38	11.62	21.70	22.16	30.43
Isoprenyl 3-acetoxyoctadecanoate (**11**)	43.73	26.16	31.13	29.56	26.20
Isoprenyl 3-acetoxy-13-eicosenoate	1.20	0.04	0.98	0.10	0.01
Isoprenyl 3-acetoxyeicosanoate	0.52	0.00	0.18	0.01	0.00

Table S4. Relative proportions of compounds occurring at least two times in five samples of androconia of male *I. s. derasa.* Column heads show internal sample code number and sample collection number (see Table S2).

	ISD-1 LdeS11-81	ISD-2 LdeS11-82	ISD-3 LdeS11-1294	ISD-4 LdeS11-1293	ISD-6 LdeS11-270
ß-Elemene	0.01	0.19	0.00	0.02	0.08
Elemol/Hedycaryol isomer	0.03	0.06	0.00	0.00	0.02
α-Elemol (**8**)	1.85	2.88	0.11	0.49	1.74
Elemol/Hedycaryol isomer	0.01	0.02	0.00	0.01	0.00
Hexadecanoic acid	0.25	0.03	0.02	0.00	0.00
7-Heneicosene	13.97	0.15	4.25	0.00	0.00
Heneicosane	0.54	0.02	0.10	0.00	0.00
Octadecenoic acid	3.69	2.20	1.71	0.62	0.00
Ticosane	0.44	0.07	0.14	0.01	0.04
11-Methyltricosane	4.04	0.60	1.30	0.00	0.06
Eicosenoic acid	0.96	0.08	0.08	0.00	0.00
Isoprenyl octadecadienoate	0.01	0.30	0.00	0.02	0.15
Isoprenyl 9-octadecenoate	0.76	4.81	0.36	4.90	8.27
Isoprenyl 11-octadecenoate	0.00	0.00	0.00	0.01	0.02
Isoprenyl octadecanoate	0.03	0.32	0.01	0.03	0.01
Isoprenyl 3-acetoxy-11-hexadecenoate	0.44	0.38	0.40	0.10	0.10
Isoprenyl 3-acetoxyhexadecanoate	1.32	0.92	0.99	0.51	0.30
Pentacosane	0.12	0.03	0.03	0.01	0.13
Isoprenyl (2*E*,11*Z*)-2,11-octadecadienoate	12.63	4.60	0.14	0.00	3.31
Isoprenyl (2*E*,13*Z*)-2,13-octadecadienoate	0.28	0.92	0.01	0.00	0.33
Isoprenyl 2-octadecenoate	0.55	1.96	0.05	0.04	0.88
11- and 13-Methylpentacosane	0.02	0.03	0.05	0.00	0.00
Isoprenyl 3-hydroxy-11-octadecenoate	1.10	5.02	4.22	4.11	2.83
Isoprenyl 3-hydroxy-13-octadecenoate (**24**)	0.08	0.24	0.40	0.08	0.07
Isoprenyl 3-hydroxyoctadecanoate	0.98	1.64	2.41	1.32	1.31
Isoprenyl (*Z*)-3-acetoxy-11-octadecenoate	22.72	44.66	37.34	45.28	44.78
Isoprenyl (*Z*)-3-acetoxy-13-octadecenoate (**12**)	3.87	5.01	14.67	9.73	11.37
Isoprenyl 3-acetoxyoctadecanoate (**11**)	22.32	16.01	25.44	25.24	19.87
Isoprenyl 3-hydroxy-13-eicosenoate	0.00	0.00	0.00	0.45	0.10
Isoprenyl 3-acetoxy-13-eicosenoate	6.65	6.77	5.67	6.81	4.25
Isoprenyl 3-acetoxyeicosanoate	0.35	0.12	0.09	0.17	0.02

3.2 Experimental conditions

Commercially available chemicals were used without further purification when not stated otherwise. When moisture and air-sensitive compounds were used, reactions were carried out in glass ware dried with heating under a nitrogen atmosphere. Solvents for chromatography were distilled before use. Solvents were dried by conventional methods if needed. ^{1}H-NMR and ^{13}C-NMR spectra were recorded with the instruments DPX-200 (200 MHz for ^{1}H and 50 MHz for ^{13}C) and DRX-400 (400 MHz for ^{1}H and 100 MHz for ^{13}C) from Bruker. Tetramethylsilane was used as internal standard. The multiplicities of the protons are indicated by singlet (s), doublet (d), triplet (t), quartet (q), quintet (quint), sextet (sext) or multiplet (m). The multiplicities of the carbon atoms are given equivalently as quaternary (s), tertiary (d), secondary (t) or primary (q). Wide signals are indicated by b before the multiplicity.
Gas chromatographic investigations were performed with an HP 7820A with FID detector and an ALS 7683 autosampler (Agilent). A fused silica capillary column HP-5 MS (Agilent, 30 m, 0.25 mm, 0.25 µm film thickness) was used. Hydrogen was used as carrier gas. GC/MS investigations of synthetic samples were performed with a HP 6890/MSD 5973 combination (Agilent) in EI mode (70 eV). For chromatographic separations a fused silica capillary BPX-5 (SGE Inc., 25 m, 0.22 mm OD; 0.25 µm film thickness) was used. Helium was used as carrier gas. High-resolution MS data were obtained with an Agilent 6890 gas chromatograph coupled to a JMS-T100GC (GCAccuTOF, JEOL) equipped with a ZB5-MS (Phenomenex, 30 m × 0.25 mm i.d. × 0.25 µm) column.
Natural extracts were analyzed using either an HP 7890A/MSD 5975 combination and an ALS 7683 Autosampler (Agilent) or a HP 7890B/MSD 5977 combination (Agilent) and an MPS auto-sampler (Gerstel) in EI mode (70 eV). For gas chromatographic separations a fused silica capillary column HP5-MS (Agilent, 30 m, 0.25 mm OD, 0.25 µm film thickness) was used. Helium was used as carrier gas. The samples were concentrated to approx. 20 µL in a nitrogen stream before injection. The starting temperature of the oven was 50 °C, which was maintained for 5 minutes. The oven was then heated to 320 °C at 5 °C/minute and the temperature was maintained for another 10 minutes.
Determination of the enantiomeric compositions were performed on a chiral Hydrodex β-6TBDM type (Macherey & Nagel, 25 m, 0.25 mm OD, 0.25 µm film thickness) GC phase using the combination HP 7890B/MSD 5977 from Agilent and an MPS Autosampler from Gerstel. The mass spectrometer was operated in EI-mode at 70 eV. Helium was used as carrier gas.
The starting temperature was 160 °C, which was maintained for 360 minutes. Subsequently, the temperature was raised to 220 °C at 25 °C/min.
IR spectra were recorded with the Tensor 27 instrument from Bruker using the diamond ATR technique. GC/DD-IR analyses was performed using a Dani Instruments DiscovIR IR detector coupled to an Agilent Technologies 7890B gas chromatograph. Gas chromatographic separations were performed with a fused silica capillary column HP5-MS (Agilent, 30 m, 0.25 mm OD, 0.25 µm film thickness). Helium was used as carrier gas and residual water was removed using a conventional in-line water trap for gases.

The positions of the absorption bands are indicated as wave numbers in cm^{-1}. The intensities are marked with s (strong), m (medium) and w (weak), broadened bands are additionally marked with br (broad).
UV/VIS-Spectra were measured with Cary 100 Bio spectrometer (Varian). The wavelength λ of the absorption maxima is listed in nm, the extinction ε in cm^{-2}•$mmol^{-1}$.
Products were purified by column chromatography on silica gel (Fluka, silica gel 60, particle size 0.040-0.063 mm, mesh 230-440 ASTM). Various solvent mixtures of pentane and diethyl ether were used as indicated. Thin layer chromatography was performed with silica gel foil Polygram SIL G/UV254 (Macherey & Nagel). Detection was performed by UV (254 nm), potassium permanganate immersion, vanillin immersion, molybdatophosphoric acid immersion or by Vaughn's reagent.
The rotation value of optically active compounds was determined with a Propol Digital Automatic Polarimeter from Dr. Kernchen, measured in a 1 cm cuvette at a wavelength of 589 nm.

3.3 Microderivatizations

Transesterification

Sodium methanolate in abs. methanol (0.2 mL, 0.5M) was added to about 20 µL of a natural extract or 0.5 mg synthetic material in 0.1 mL pentane. The sodium methanolate solution was prepared by adding freshly cut sodium pieces to abs. methanol (caution: H_2 gas evolution). The mixture was heated to 60°C for 10 minutes in a closed cup vial and then mixed with glacial acetic acid (10 µL) and water (0.5 ml). The aqueous phase was extracted twice with pentane (0.5 mL) and the organic phase way dried with a small amount of $MgSO_4$. The sample was concentrated in a stream of nitrogen.

DMDS adducts

To about 20 µL of a natural extract in 20 µL mL pentane was added dimethyl disulfide (50 µL) and a solution of iodine in diethyl ether (5 µL, 0.24 M). The mixture was heated to 40 °C for 15 h in a closed vial. Pentane (200 µL) was added, and the mixture was washed with 5% $Na_2S_2O_3$-solution. The organic phase was separated, dried with $MgSO_4$ and concentrated in a stream of nitrogen.

3.4 Synthesis

Hexadecanal (14)

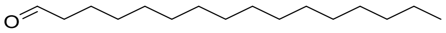

According to the procedure of More and Finney [S2], Hexadecanol (**13**, 5.00 g, 20.62 mmol, 1 eq) was dissolved in ethyl acetate (150 ml) and IBX (16.8 g, 60 mmol) was added. The solution was heated to reflux until the alcohol was completely converted (2.5 to 3.25 hours). After cooling, the solid was filtered through a frit and washed three times with ethyl acetate. The solvent was removed under vacuum and the crude

product purified by column chromatography with 20:1 pentane/*tert*-butyl methyl ether (TBME). A colorless waxy solid was obtained (4.9 g, 20.4 mmol, 99%).

DC (40:1 pentane/TBME): R_f = 0.45. ^{1}H-NMR (400 MHz, $CDCl_3$): δ [ppm] = 9.76 (t, 1H, H-1); 2.41 (dt, 2H, $^3J_{1H,2H}$ = 1.9 Hz, $^3J_{2H,3H}$ = 7.3 Hz, H-2); 1.63 (quint, 2H, ^3J = 7.3 Hz, CH_2); 1.35-1.20 (m, 24H, CH_2); 0.88 (t, 3H, ^3J = 6.9 Hz, H-16). ^{13}C-NMR (100 MHz, $CDCl_3$): δ [ppm] = 203.0 (C-1); 43.0 (t, C-2); 32.0 (t, C-14); 29.8-29.7 (t, 6C); 29.6 (t); 29.5 (t); 29.4 (t); 29.2 (t); 22.7 (t); 22.1 (t); 14.2 (q, C-16). GC/MS (EI70 eV): *m*/*z* (%) = 240 [M^+] (0.2); 222 (1); 196 (3); 194 (3); 166 (2); 96 (24); 82 (35); 69 (23); 67 (24); 57 (50); 55 (52); 43 (77); 41 (100). IR: $\tilde{\nu}$ [cm^{-1}] = 2957(w); 2914(s); 2848(s); 2751(w); 1704(s); 1470(m); 1392(w); 1372(w); 895(w); 716(w); 698(w); 657(w).

Ethyl 3-oxooctadecanoat (16)

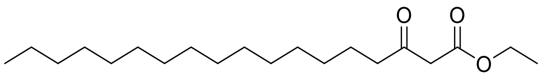

According to the general procedure for the synthesis of β-keto esters by Holmquist and Roskamp [S3], ethyl diazoacetate (**15**, 2.2 mL, 20.96 mmol, 1.05 eq) was dissolved in dry dichloromethane (40 mL) under inert gas and tin(II) chloride (0.379 g, 1.99 mmol, 0.1 eq) was added. A few drops of a solution of hexadecanal (4.9 g, 20.37 mmol, 1 eq.) in dry dichloromethane (10 mL) were slowly added. After the start of the reaction (gas formation) the remaining hexadecanal solution was slowly added over 10 minutes. The solution was stirred for 3 h at room temperature. Then the mixture was poured into 100 mL sat. NaCl solution. The resulting emulsion was destroyed with KOH. The phases were separated and the aqueous phase was extracted three times with dichloromethane. The combined organic phases were dried and concentrated over Na_2SO_4. The raw product was purified by column chromatography with a 20:1 to 10:1 pentane/TBME gradient. Yield: 4.67 g (14.3 mmol, 70%) of a white, waxy solid

DC (20:1 pentane/TBME): R_f = 0.34.

^{1}H-NMR (400 MHz, $CDCl_3$): δ [ppm] = 4.20 (q, 2H, $^3J_{Ethyl}$ = 7.1 Hz, OCH_2CH_3); 3.43 (s, 2H, $COCH_2COO$); 2.53 (t, 2H, $^3J_{4H\text{-}5H}$ = 7.4 Hz, H-4); 1.65-1.53 (m, 2H, H-5); 1.27 (q, 3H, $^3J_{Ethyl}$ = 7.1 Hz, OCH_2CH_3); 1.33-1.21 (m, 24H, CH_2); 0,88 (t, 3H, $^3J_{17,18}$ = 6.9 Hz, CH_3CH_2O). ^{13}C-NMR (100 MHz, $CDCl_3$): δ [ppm] = 203.0 (s, C-3); 167.3 (s, C-1); 61.3 (t, $COOCH_2CH_3$); 49.3 (t, C-2); 43.0 (t, C-4); 31.9 (t, C-6); 29.7-29.6 (t, 6C); 29.5 (t); 29.4 (t); 29.3 (t); 29.0 (t); 23.45 (t, C-5); 22.7 (t, C-17); 14.10 (d, $COOCH_2CH_3$); 14.08 (d, C-18). GC/MS (EI, 70 eV): *m*/*z* (%) = 326 [M^+] (0.5); 269 (4); 253 (3); 130 (7); 111 (7); 109 (8); 104 (8); 97 (17); 95 (18); 83 (22); 81 (19); 71 (13); 69 (25); 67 (17); 57 (54); 55 (53); 43 (100); 41 (96). IR: $\tilde{\nu}$ [cm^{-1}] = 2983 (w); 2956 (w); 2917 (s); 2849 (s); 1739(s); 1710(s); 1468 (m); 1410 (m); 1366 (m); 1323 (m); 1253(m); 1162(m); 1076 (m); 1036 (m); 943 (w); 719 (m); 653 (w); 558 (w). UV/VIS (DCM): λ_{max} [nm] (lg ε) = 247 (2.77); 224 (2.53).

Methyl 3-hydroxyoctadecanoate (17)

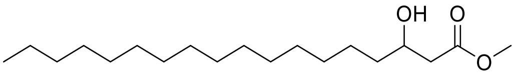

Ketoester **16** (750 mg, 2.3 mmol, 1 eq) was dissolved in methanol (2 mL) and $NaBH_4$ (44 mg, 1.15 mmol, 0.5 eq) was slowly added while stirring. The reaction mixture was stirred for 12 h and then concentrated *in vacuo*. The residue was mixed with water and extracted three times with TBME. The combined organic layers were washed with brine and dried with $MgSO_4$. The solvent was removed under vacuum and the product **17** was obtained in quantitative yield (723 mg, 2.3 mmol) as a yellowish, waxy solid. During the reaction transesterification to the methyl ester was observed.
DC (3:1 pentane/TBME): R_f = 0.33.); ^{1}H-NMR (200 MHz, $CDCl_3$): δ [ppm] = 4.04 (m, 1H, H-3), 3.72 (s, 3H, OCH_3); 2.86 (bs, 1H, OH); 2.52 (dd, 1H, $^3J_{2H,3H}$ = 3.1 Hz, $^2J_{2H,2Hb}$ = 16.4 Hz, H-2); 2.41 (dd, 1H, $^3J_{2H,3H}$ = 9.0 Hz, $^2J_{2H,2Hb}$= 16.4 Hz, H-2); 1.70- 1.48 (m, 4H); 1.47-1.38 (m, 3H); 1.37-1.16 (m, 28H); 0.88 (t, 3H, $^3J_{17H,18H}$= 6.9 Hz, H-18). ^{13}C-NMR (100 MHz, $CDCl_3$): δ [ppm] = 173.5 (s, C-1); 68.0 (C-3); 51.7 (q, COO*C*H_3); 41.1 (t, C-2); 36.5 (t, C-4); 31.9 (t, C-16); 29.7-29.6 (t, 6C); 29.6 (t); 29.5 (t); 29.4 (t); 29.3 (t); 25.5 (t, C-5); 22.7 (t, C-17); 14.1 (d, C-18). GC/MS (EI, 70 eV): *m*/*z* (%) = 314 [M^+] (0.1); 313 (0.1); 296 (1); 264 (4); 222 (3); 111 (3); 103 (100); 97 (7); 74 (17); 71 (18); 57 (12); 55 (13); 43 (27). IR: $\tilde{\nu}$ [cm^{-1}] = 3375 (br); 2955 (w); 2916 (s); 2849 (s); 1732 (m); 1466 (w); 1439 (w); 1196 (w); 1173 (m); 1081 (w); 990 (w); 721 (w).

3-Methyl-3-butenyl 3-hydroxyoctadecanoate (18)

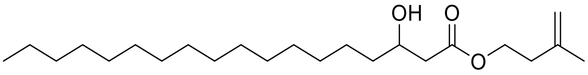

According to the transesterification method of Otera et al. [S4], ester **17** (0.37 g, 1.2 mmol, 1 eq.) was dissolved in 10 mL 3-methyl-3-buten-1-ol. Dibutyltin oxide (0.03 g, 0.12 mmol, 0.1 eq) was added as catalyst. The mixture was heated to reflux (140 °C) for 48 hours, followed by washing with sat. sodium bicarbonate solution and three times extraction with ethyl acetate. The combined organic phases were washed with 10% KF solution and brine and dried over magnesium sulfate. The solvent was removed under vacuum. The crude product was purified by column chromatography with a 3:1 pentane/diethyl ether mixture. Yield: 375 mg (0.95 mmol, 78%) of a white, waxy solid.
DC (3:1 pentane/diethyl ether): R_f = 0.4. ^{1}H-NMR (400 MHz, $CDCl_3$): δ [ppm] = 4.83-4.81 (m, 1H, H-4'); 4.76-4.74 (m, 1H, H-4'); 4.24 (t, 2H, $^3J_{1'H,2'H}$= 6.7 Hz, H-1'); 4.03-3.94 (m, 1H, H-3); 2.90 (bs, 1H, OH); 2.51 (dd, 1H, $^3J_{2H,3H\ erythro}$= 3.3 Hz, $^2J_{2H,2Hb}$= 16.3 Hz, H-2); 2.41 (dd, 1H, $^3J_{2H,3H}$ = 9.0 Hz, $^2J_{2H,2Hb}$= 16.3 Hz, H-2); 2.36 (t, 2H, $^3J_{1'H,2'H}$= 6.7 Hz, H-2'); 1.76 (s, 3H, CH_3-3'); 1.57-1.47 (m, 2H); 1.46-1.38 (m, 2H); 1.34-1.20 (m, 24H); 0.88 (t, 3H, $^3J_{17H,18H}$= 6.9 Hz, H-18). ^{13}C-NMR (100 MHz, $CDCl_3$): δ [ppm] = 173.0 (s, C-1); 141.7 (s, C-3'); 112.4 (s, C-4'); 68.0 (d, C-3); 62.7 (t, C-1'); 41.4 (t, C-2); 36.6 (t, C-2'); 36.5 (t, C-4); 31.9 (t, C-16); 29.7-29.6 (t, 6C); 29.6 (t); 29.56 (t); 29.53 (t); 29.4 (t); 25.5 (t, C-5); 22.7 (t, C-17); 22.4 (d, C-3'); 14.1 (d, C-18). GC/MS (EI, 70 eV): *m*/*z* (%) = 368 [M^+] (0.1); 350 (1); 299 (3); 283 (7); 265 (5);

252 (9); 250 (5); 239 (20); 157 (19); 97 (14); 83 (14); 69 (100); 68 (93); 57 (17); 55 (22); 53 (14); 43 (26); 41 (32). IR: $\tilde{\nu}$ [cm^{-1}] = 3375 (br); 3078 (w); 2956 (w); 2916 (s); 2849 (s); 1730 (m); 1651 (w); 1467 (w); 1406 (w); 1295 (w), 1174 (s); 1079 (s); 889 (m); 803 (w); 721 (w). UV/VIS (DC-M): λ_{max} [nm] (lg ε) = 227 (2.3).

3-Methyl-3-butenyl 3-acetoxyoctadecanoate (11)

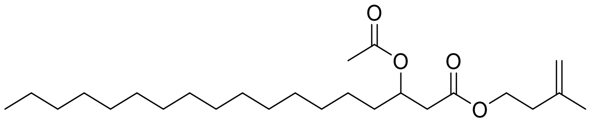

Acetylation was performed using a modified general procedure [S5]. Ester **18** (426 mg, 1.16 mmol, 1 eq) was dissolved in dry dichloromethane (4.5 ml) under nitrogen gas atmosphere. The mixture was cooled to 0°C and mixed with pyridine (138 mg, 1.74 mmol) and DMAP (14 mg, 0.12 mmol). Then acetic anhydride (142 mg, 1.4 mmol) was slowly added. The reaction mixture was warmed to room temperature and stirred until complete conversion was achieved. Brine was added, and the phases were separated. The aqueous phase was extracted three times with dichloromethane and the combined organic phases dried with Na_2SO_4. The solvent was removed under vacuum and the raw product was purified by column chromatography with a 5:1 pentane/diethyl ether solvent mixture. Yield: 19 mg (0.78 mmol, 67%) of a white, waxy solid.
DC (5:1 pentane/diethyl ether): R_f = 0.4.
^{1}H-NMR (400 MHz, $CDCl_3$): δ [ppm] = 5.20 (ddt, 1H, $^3J_{3H,4H}$= 7.1 Hz; $^3J_{2H,3H}$= 7.3 Hz, $^3J_{2Hb\text{-}3H}$= 5.5 Hz, H-3); 4.83-4.79 (m, 1H, H-3'); 4.75-4.72 (m, 1H, H-3'); 4.20 (t, 2H, $^3J_{1'H,2'H}$= 6.9 Hz, H-1'); 2.58 (dd, 1H, $^3J_{2H,3H}$= 7.3 Hz, $^2J_{2H,2Hb}$= 15.5 Hz, H-2); 2.52 (dd, 1H, $^3J_{2H,3H}$= 5.2 Hz, $^2J_{2H,2Hb}$= 15.5 Hz, H-2); 2.33 (t, 2H, $^3J_{1'H,2'H}$= 6.8 Hz, H-2'); 2.02 (s, 3H, CH_3-C=O); 1.75 (s, 3H, CH_3-3'); 1.57-1.47 (m, 2H); 1.46-1.38 (m, 2H); 1.34-1.20 (m, 24H); 0.88 (t, 3H, $^3J_{17H,18H}$= 6.9 Hz, H-18). ^{13}C-NMR (100 MHz, $CDCl_3$): δ [ppm] = 170.4 (s, C-1); 170.3 (s, CH_3C=O); 141.5 (s, C-3'); 112.3 (s, C-4'); 70.5 (d, C-3); 62.8 (t, C-1'); 39.2 (t, C-2); 36.6 (t, C-2'); 34.0 (t, C-4); 31.9 (t, C-16); 29.7-29.6 (t, 7C); 29.5 (t); 29.4 (t); 29.3 (t); 25.1 (t, C-5); 22.7 (t, C-17); 22.4 (d, CH_3-3'C); 21.1 (d, CH_3C=O); 14.1 (d, C-18). GC/MS (EI, 70 eV): *m*/*z* (%) = 410 (0.6); 351 (8); 350 (4); 343 (5); 299 (5); 283 (18); 265 (36); 239 (3); 199 (3); 179 (4); 128 (8); 111 (11); 97 (10); 83 (8); 69 (53); 68 (100); 61 (2); 57 (12); 55 (26); 43 (71); 41 (43). IR: $\tilde{\nu}$ [cm^{-1}] = 3077 (w); 2923 (s); 2853 (m); 1741 (s); 1651 (w); 1462 (w); 1373 (w); 1235 (s), 1173 (m); 1025 (m); 891 (w); 721 (w). UV/VIS (DC-M): λ_{max} [nm] (lg ε) = 229 (2.3).

9-Bromo-1-nonanol

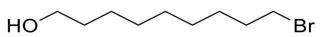

According to the procedure of Chong et al. [S6], nonane-1,9-diol (**19**, 2.99 g, 18.7 mmol, 1 eq.) was dissolved in toluene (860 mL) and 48% aqueous HBr was added (2.6 mL, 22.42 mmol, 1.2 eq.). The reaction mixture was heated to reflux for 24 h. Again 48% aqueous HBr (1.1 mL, 9.35 mmol, 0.5 eq.) was added to the reaction mixture and heating continued for another 24 hours. The reaction was stopped by addition of 1M NaOH solution, the phases were separated and the aqueous phase was extracted

three times with toluene. The combined organic extracts were dried with $MgSO_4$ and the solvent was removed under vacuum. The resulting crude product was purified with a 1:1 pentane/diethyl ether solvent mixture by column chromatography. 9-Bromo-1-nonanol (3.28 g, 14.79 mmol) was obtained as a colorless liquid in 79 % yield.
DC (1:1 pentane/diethyl ether): R_f = 0.39. ^{1}H-NMR (400 MHz, $CDCl_3$): δ [ppm] = 3.64 (t, 2H, $^3J_{1H,2H}$= 6.6 Hz, H-1); 3.41 (t, 2H, $^3J_{8H\text{-}9H}$= 6.8 Hz, H-9); 1.87 (tt, 2H, $^3J_{7H\text{-}8H}$= 7.2 Hz, $^3J_{8H\text{-}9H}$= 6.8 Hz H-8), 1.57 (quint, 2H, $^3J_{1H,2H}$ = 6.6 Hz, $^3J_{2H,3H}$ = 6.6 Hz, H-2), 1.49-1.41 (m, 2H); 1.41-1.31 (m, 8H). ^{13}C-NMR (100 MHz, $CDCl_3$): δ [ppm] = 63.0 (t, C-1); 34.0 (t, C-9); 32.8 (t, C-7); 32.8 (t, C-2); 29.4 (t), 29.3 (t); 28.7 (t); 28.1 (t); 25.7 (t). GC/MS (EI, 70 eV): *m/z* (%) = 204/206 (0.2); 176/178 (10); 162/164 (20); 148/150 (34); 135/137 (52); 107/109 (7); 97 (78); 83 (53); 69 (93); 55 (100); 41 (54). IR [cm^{-1}] = 3421 (bw); 3363 (bw); 2920 (s); 2891 (m); 2852 (s); 1464 (w); 1053 (m); 1012 (m); 977 (w); 726 (m); 645 (s).

9-Bromononanal (20)

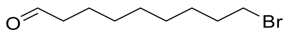

9-Bromononanol (4.89 g, 21.91 mmol, 1 eq.) was oxidized as described for compound **13**. The resulting crude product was purified by column chromatography with a 10:1 pentane/diethyl ether solvent mixture. Aldehyde **20** was obtained (4.34 g, 19.72 mmol, 90%) as a colorless oil.
DC (10/1 pentane/diethyl ether): R_f = 0.31. ^{1}H-NMR (200 MHz, $CDCl_3$): δ [ppm] = 9.77 (t, 1H, $^3J_{1H,2H}$ = 2.0 Hz, H-1); 3.41 (t, 2H, $^3J_{9H\text{-}8H}$ = 8.1 Hz, H-9); 2.43 (dt, 2H, $^3J_{2H,3H}$ = 7.3 Hz, $^3J_{1H,2H}$ = 2.0 Hz, H-2); 1.84 (tt, 2H, $^3J_{9H\text{-}8H}$= 8.1 Hz, $^3J_{8H\text{-}7H}$=7.0 Hz, H-7); 1.68-1.58 (m, 2H, H-3); 1.47-1.37 (m, 2H, H-4); 1.37-1.29 (m, 6H). ^{13}C-NMR (100 MHz, $CDCl_3$): δ [ppm] = 202.8 (d, C-1); 43.8 (t, C-2); 33.9 (t, C-9); 32.7 (t); 29.1 (t); 29.0 (t); 28.5 (t); 28.1 (t); 22.0 (t). GC/MS (EI, 70 eV): *m/z* (%) = 202/204 (3); 192/194 (2); 176/178 (25); 135/137 (9); 123 (17); 97 (60); 81 (49); 69 (36); 67 (33); 55 (100); 44 (58); 41 (67). HR-MS: 202.0353 (M^+-H_2O, calcd. 202.0357 for $C_9H_{15}Br_1$). IR: [cm^{-1}] = 2929 (s); 2855 (m); 2718 (w); 1723 (s); 1462 (w); 1246 (w); 935 (w); 724 (w); 643 (m); 561 (m).

(*Z*)-14-Bromo-5-tetradecene (21)

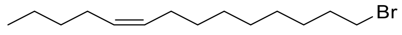

By use of a modified procedure by Bestmann et al. [S7], diisopropylamine (2.67 g, 26.4 mmol, 1.2 eq.) was dissolved in dry THF (50 mL) under nitrogen gas atmosphere and cooled to -78 °C. The solution was slowly mixed at 78 °C with n-BuLi solution (20.6 mL, 1.6M in hexane, 39 mmol, 1.5 eq.) and stirred for 15 minutes at the same temperature. This freshly prepared LDA solution was added dropwise to a 0 °C cold solution of pentyltriphenylphosphonium bromide (10.00 g, 24.2 mmol, 1.1 eq.) in dry THF (450 mL) and stirred for one hour at the same temperature. The solution was then cooled down again to -78 °C, **20** (3.85 g, 17.4 mmol, 1 eq.) was added dropwise, and the mixture was stirred overnight at the same temperature. The reaction was stopped by adding H_2O. The aqueous phase was extracted three times with pentane, the combined organic phases were washed with brine and dried over $MgSO_4$. The resulting

crude product was purified by column chromatography with pentane, yielding **21** in a 9:1 *Z*/*E*-ratio (4.00 g, 14.6 mmol, 84%) as colorless oil.

DC (pentane): R_f = 0.8. ^{1}H-NMR (400 MHz, $CDCl_3$): δ [ppm] = 5.40-5.30 (m, 2 H, H-5, H-6); 3.41 (t, 2 H, $^{3}J_{1H,2H}$ = 6.8 Hz, H-14); 2.08-1.98 (m, 4 H, H-4, H-7); 1.86 (quint, 2 H, ^{3}J = 6.8 Hz, H-13); 1.47–1.23 (m, 14 H); 0.90 (t, 3 H, $^{3}J_{13H\text{-}14H}$ = 7.0 Hz, H-14). ^{13}C-NMR (100 MHz, $CDCl_3$): δ [ppm] = 129.9 (d); 129.8 (d); 34.0 (t, C-14); 32.8 (t, C-13), 32.0 (t, 1 C); 29.7 (t, 1 C); 29.3 (t); 29.2 (t); 28.7 (t); 28.1 (t); 27.1 (t); 26.9 (t); 22.3 (t); 14.0 (q, C-1). GC/MS (EI, 70 eV): *m/z* (%) = 274/276 $[M]^+$ (20); 246/248 (2); 190/192 (4); 176/178 (4); 162/164 (21); 148/150 (36); 137 (17); 123 (13); 111 (25), 97 (68); 83 (69); 69 (83); 55 (100); 41 (61). HR-MS: 274.1302 (M^+, calcd. 274.1296 for $C_{14}H_{27}Br_1$). IR [cm^{-1}] = 3004 (w); 2924 (s); 2854 (m); 1462 (w); 1247 (w); 968 (w); 722 (w); 647 (w); 563 (w). UV/VIS (pentane): (lg ε) = 195 (3.7).

Ethyl (3*R*,13*Z*)-3-hydroxy-13-octadecenoate (23)

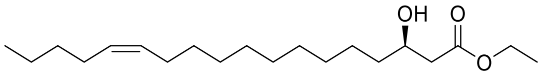

A suspension of magnesium turnings (0.119 g, 4.895 mmol, 2.55 eq.) in dry THF (0.50 mL) was mixed with 1,2-dibromoethane (45.6 mg, 0.243 mmol, 0.13 eq.) under inert gas and allowed to stir for 20 minutes. In order to remove the resulting magnesium salts, the supernatant was then removed with a syringe, and a small amount of iodine was added to the Mg. A solution of **21** (1.267 g, 4.61 mmol, 2.4 eq.) in abs. THF (6.49 mL) was prepared. First, 0.75 mL of this solution were added to the activated magnesium to start the reaction. Then the remainder of the solution of **21** was added over a period of 30 minutes. The reaction mixture was stirred for one hour at room temperature after the initial boiling subdued.

Using the procedure developed by Huang et al. [S8], Cu(I)I (403 mMol, 2.11 mmol, 1.1 eq.) was suspended in dry THF (7.03 mL) under inert gas atmosphere in a separate reaction vessel and mixed with LiCl (178 mg, 4.22 mmol, 2.2 eq.), freshly dried under vacuum at 150 °C for one hour. The solid was dissolved and a clear yellow solution was formed. This solution was cooled to -30 °C and slowly mixed with the previously prepared organometallic solution. The solution was stirred for 50 minutes at the same temperature; after a short time it turned bluish-black. A solution of enantiomerically pure epoxide (*S*)-**22** (0.250 g, 1.92 mmol, 1 eq.) in dry THF (1.00 mL) was then slowly added to the cuprate solution. The reaction mixture was stirred at -30 °C for one hour. The cooling was removed and stirring continued for 12 hours. The reaction was stopped by adding saturated NH_4Cl solution, and the phases were separated. The aqueous phase was extracted three times with diethyl ether, the combined organic phases were washed with saturated NaCl solution and dried over $MgSO_4$. The solvent was removed under vacuum. The non-polar by-products were first separated by column chromatography with pentane and the pure product was eluted with diethyl ether. Ester **23** was obtained as colorless oil (497 mg, 1.52 mmol, 79%).

$[\alpha]_D^{22.5}$ = +2.8 ($CHCl_3$, *c* = 1). ^{1}H-NMR (400 MHz, $CDCl_3$): δ [ppm] = 5.40-5.31 (m, 2H, H-13, H-14); 4.20 (q, 2H, ^{3}J = 6.9 Hz, OCH_2CH_3); 4.02-3.97 (m, 1H, H-3); 2.92 (bs, 1H, O*H*); 2.50 (dd, ^{2}J =16.5 Hz, $^{3}J_{2H,3H}$ = 3.1 Hz, H-2); 2.39 (dd, ^{2}J =16.5 Hz,

$^{3}J_{2H,3H}$ = 9.0 Hz, H-2); 2.05-1.94 (m, 4H, H-12, H-14); 1.58-1.22 (m, 20H,); 1.28 (t, 3H, ^{3}J = 6.9 Hz, OCH$_2$C*H*$_3$); 0.90 (m, 3H, $^{3}J_{17H,18H}$=7.1 Hz, H-18). ^{13}C-NMR (100 MHz, CDCl$_3$): *δ* [ppm] = 173.1 (s, C-1); 129.9 (d, C-14); 129.8 (d, C-13); 68.0 (s, C-3); 60.6 (t, O*C*H$_2$CH$_3$); 41.3 (t, C-2); 36.5 (t, C-4); 32.0 (t, C-16); 29.8 (t); 29.50 (t, 4C); 29.28 (t); 27.2 (t, C-12); 26.9 (t, C-15); 25.5 (t, C-5); 22.3 (t, C-17); 14.1 (q, OCH$_2$*C*H$_3$); 14.0 (q, C-18). GC/MS (EI, 70 eV): *m/z* (%) = 326 [M]$^+$ (2); 308 (5); 263 (14); 262 (9); 251 (22); 249 (1); 235 (10); 220 (22); 164 (11); 163 (11); 150 (22) 133 (23); 123 (25); 121 (22); 117 (51); 109 (46); 95 (79); 81 (91); 69 (51); 67 (72); 55 (100); 41 (52). HR-MS: 326.2810 (M$^+$, calcd. 326.2821 for $C_{20}H_{38}O_3$). IR [cm^{-1}] = 3435 (br); 3005 (w); 2924 (s); 2854 (m); 1734 (m); 1721 (m), 1464 (w); 1373 (w); 1179 (m); 1029 (m); 721 (w). IR [cm^{-1}] = 3435 (br); 3005 (w); 2924 (s); 2854 (m); 1734 (m); 1721 (m), 1464 (w); 1373 (w); 1179 (m); 1029 (m); 721 (w). UV/VIS (lg ε) = 227 (1.9).

3-Methyl-3-butenyl (3*R*,13*Z*)-3-hydroxy-13-octadecenoate (24)

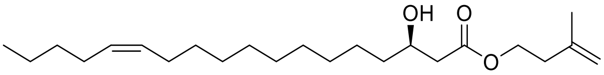

The synthesis was performed as described for compound **18**, but shortening the reaction time to 36 h. 3-Methyl-3-buten-1-ol (10 mL), (*R*)-**23** (461 mg, 14 mmol, 1 eq.), and dibutyltin oxide (35 mg, 0.14 mmol, 0.1 eq.) were used. The crude product was purified by column chromatography with a 3:1 pentane/diethyl ether mixture. Compound (*R*)-**24** was obtained as colorless liquid (336 mg, 0.92 mmol, 65%).

DC (3:1 pentane/diethyl ether): R_f = 0.30. $[\alpha]_D^{22.3}$ = +5.6. ^{1}H-NMR (400 MHz, CDCl$_3$): *δ* [ppm] = 5.40-5.31 (m, 2H, H-13, H-14); 4.83-4.81 (m, 1H, H-4'); 4.76-74 (m, 1H, H-4); 4.24 (t, 2H, ^{3}J = 6.8 Hz, H-1'); 4.02-3.97 (m, 1H, H-3); 2.87 (bd, 1H, ^{3}J = 3.4 Hz, OH); 2.50 (dd, ^{2}J =16.5 Hz, $^{3}J_{2H,3H}$ = 3.1 Hz, H-2); 2.40 (dd, ^{2}J =16.5 Hz, $^{3}J_{2H,3H}$ = 9.0 Hz, H-2); 2.36 (t, 2H, ^{3}J = 6.8 Hz, H-2'); 2.05-1.94 (m, 4H, H-12, H-15); 1.75 (s, 3H, C-3'-CH$_3$); 1.62-1.40 (m, 2H, H-4); 1.37-1.22 (m, 18H); 0.90 (m, 3H, $^{3}J_{17H,18H}$=7.1 Hz, H-18). ^{13}C-NMR (100 MHz, CDCl$_3$): *δ* [ppm] = 172.9 (s, C-1); 141.6 (s, C-3'); 129.9 (d, C-14); 129.8 (d, C-13); 112.4 (t, C-4'); 68.0 (C-3); 62.7 (t, C-1'); 41.4 (t, C-2); 36.7 (t,C-2'); 36.5 (t, C-4); 32.0 (t, C-16); 29.8 (t); 29.50 (t, 4C); 29.28 (t); 27.2 (t, C-12); 26.9 (t,C-15); 25.5 (t, C-5); 22.35 (q, C-3'-*C*H$_3$); 22.33 (t); 14.0 (q, C-18). GC/MS (EI, 70 eV): *m/z* (%) = 366 [M]$^+$ (2), 348 (2); 280 (2); 279 (3); 263 (3); 261 (2); 251 (16); 249 (10); 234 (7); 220 (5); 219 (8); 137 (7); 123 (11); 109 (17); 95 (28); 81 (28); 69 (100); 68 (35); 55 (38); 41 (36). HR-MS: 366.3112 (M$^+$, calcd. 366.3134 for $C_{23}H_{42}O_3$). IR [cm^{-1}] = 3460 (br); 3078 (w); 3004 (w); 2924 (s); 2854 (m); 1733 (m); 1651 (w); 1462 (w); 1171 (m); 977 (w); 891 (m); 721 (w). UV/VIS (lg ε) = 227 (2.2).

3-Methyl-3-butenyl (3*R*,13*Z*)-3-acetoxy-13-octadecenoate (12)

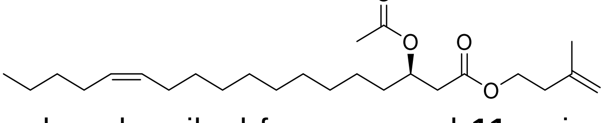

This ester was prepared as described for compound **11**, using (*R*)-**24** (296 mg, 0.8 mmol, 1 eq.) The resulting crude product was purified by column chromatography with

a 10/1 pentane/diethyl ether solvent mixture. Target compound (*R*)-**12** was obtained (243 mg, 0.6 mmol, 74%) as a colorless liquid.

DC (10:1 pentane/diethyl ether): R_f = 0.40. $[\alpha]_D^{22.4}$ = +2.7 ($CHCl_3$, *c* = 1). ^{1}H-NMR (400 MHz, $CDCl_3$): δ [ppm] = 5.40-5.31 (m, 2H, H-13, H-14); 5.19 (ddt, 1H, $^3J_{3H,4H}$ = 7.2 Hz, $^3J_{2H,3H}$ = 7.2 Hz, $^3J_{2H,3H}$ = 5.6 Hz, H-3); 4.81-4.79 (m, 1H, H-4'); 4.74-4.72 (m, 1H, H-4'H); 4.20 (t, 2H, ^3J = 6.9 Hz, H-1'); 2.58 (dd, ^2J = 15.5 Hz, $^3J_{2H,3H}$ = 7.2 Hz, H-2); 2.52 (dd, ^2J = 15.5 Hz, $^3J_{2H,3H}$ = 7.2 Hz, H-2); 2.33 (t, 2H, ^3J = 6.9 Hz, H-2'); 2.05-1.94 (m, 4H, H-12, H-15); 2.02 (s, 3H, CH_3COO); 1.75 (s, 3H, C-3'-CH_3); 1.64-1.53 (m, 2H, H-4); 1.37-1.22 (m, 18H); 0.90 (m, 3H, $^3J_{17H,18H}$ = 7.3 Hz, H-18). ^{13}C-NMR (100 MHz, $CDCl_3$): δ [ppm] = 170.4 (s, C-1); 170.3 (s, CH_3COO); 141.5 (s, C-3‘); 129.9 (d, 2C, C-13, C-14); 112.3 (t, C4'); 70.6 (C-3); 62.8 (t, C-1‘); 39.2 (t, C-2); 36.6 (t, C-2'); 34.0 (t, C-4); 32.0 (t); 29.8 (t); 29.50 (t, 2C); 29.46 (t); 29.36 (t); 29.28 (t); 27.2 (t, C-12); 26.9 (t, C-15); 25.1 (t, C-5); 22.5 (q, C-3'-CH_3); 22.3 (t, C-17); 21.1 (d, CH_3COO); 14.0 (q, C-18).

GC/MS (EI, 70 eV): *m*/*z* (%) = 408 $[M]^+$ (1), 348 (7); 280 (6); 279 (7); 263 (19); 251 (8); 220 (9); 219 (11); 137 (11); 123 (15); 109 (21); 95 (34); 81 (37); 69 (100); 68 (46); 61 (2); 55 (39); 43 (38); 41 (34). HR-MS: 348.3001 (M^+-CH_3COOH, calcd. 348.3028 for $C_{23}H_{40}O_2$). IR [cm^{-1}] = 3078 (w); 3004 (w); 2925 (m); 1651 (w); 1740 (s); 1651 (w); 1459 (w); 1373 (w); 1235 (s); 1172 (m); 1025 (m); 976 (w); 891 (w); 722 (w). UV/VIS (lg ε) = 227 (2.1).

Methyl 3-hydroxy-13-octadecenoate (25)

For the determination of the absolute configuration of natural **12** racemic material was needed to prove separation. Therefore, *rac*-**12** was synthesized by the route described above, starting with a racemic epoxide. Because separation of the **12** was not achieved, a conversion into the methyl ester **25** was performed. This transesterification was performed as described above in section 3.3

4. IR spectra of natural compounds

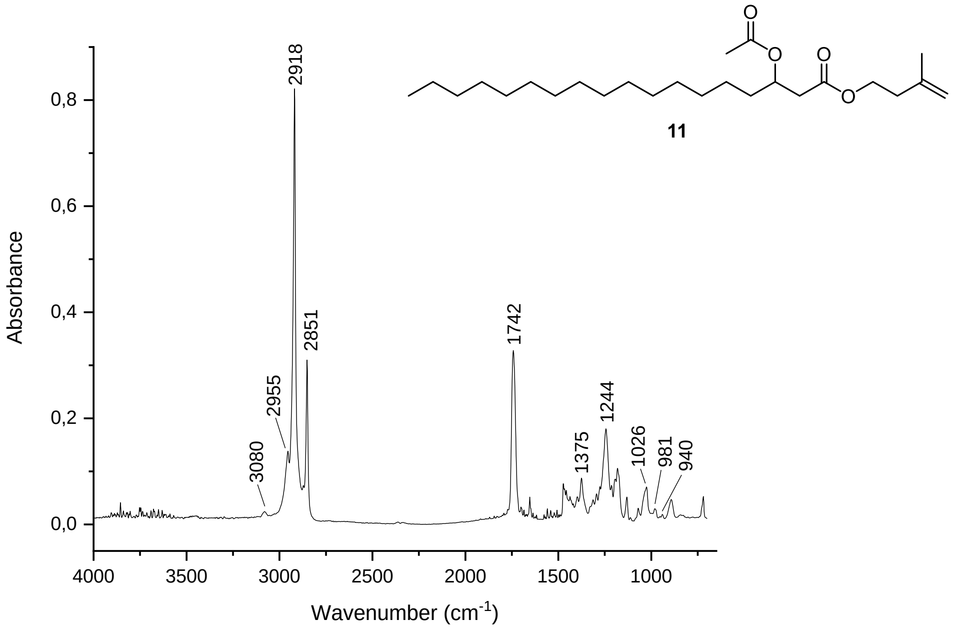

Figure S9. IR-spectrum of natural isoprenyl 3-acetoxyoctadecanoate (11) obtained by GC/DD-IR.

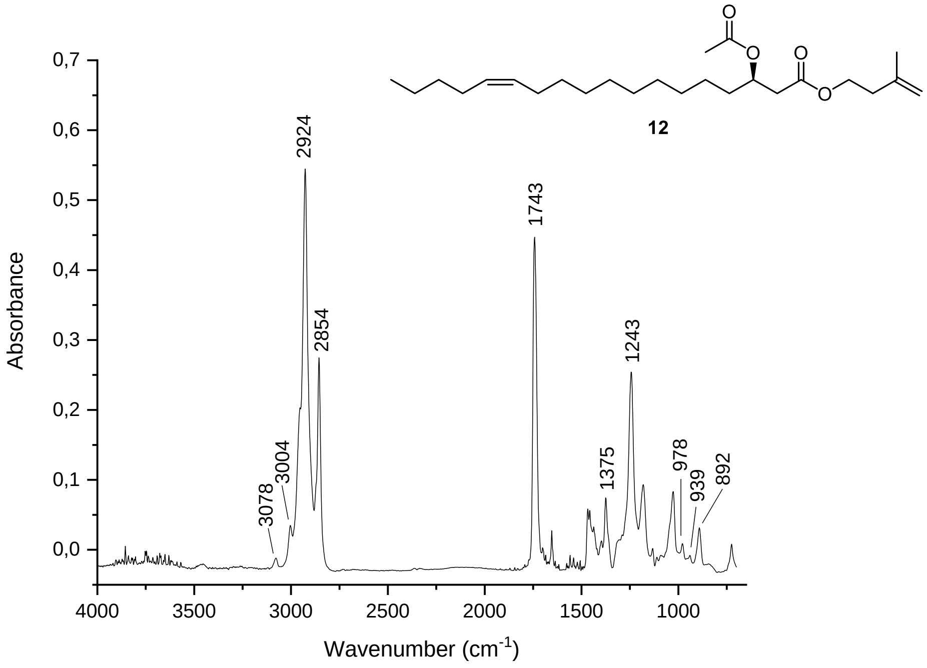

Figure S10. IR-spectrum of natural isoprenyl (3*R*,13*Z*)-3-acetoxy-13-octadecenoate (12) obtained by GC/DD-IR.

5. NMR spectra

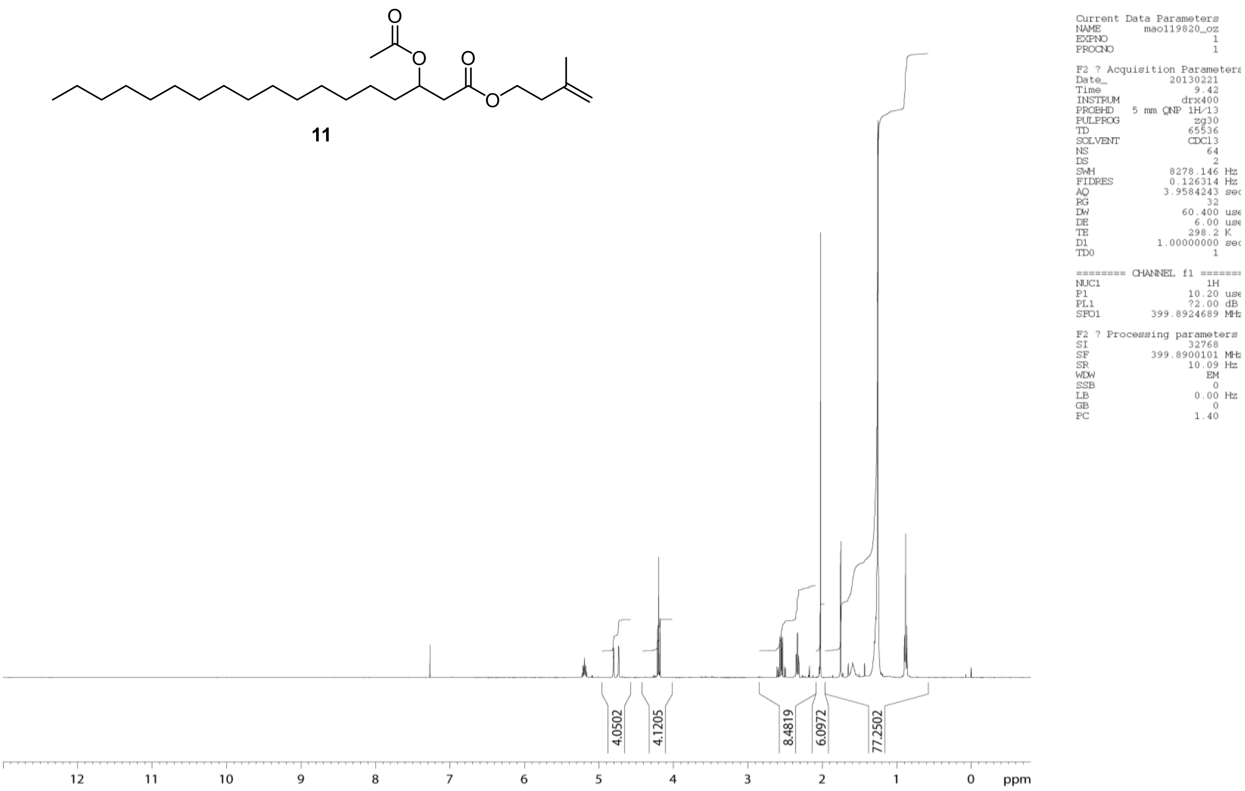

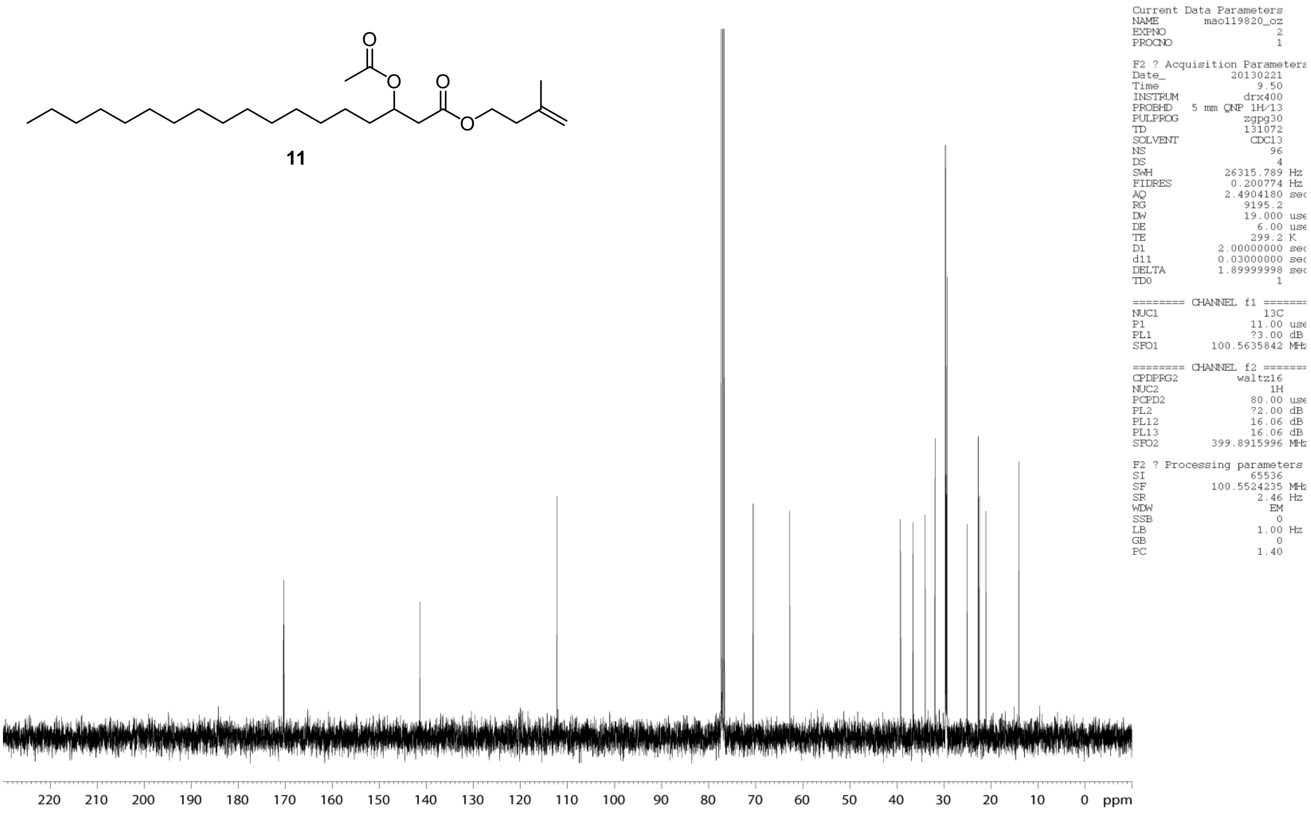

Figure S11. ^{1}**H- and** ^{13}**C-NMR spectra of isoprenyl 3-acetoxyoctadecanoate (11)**

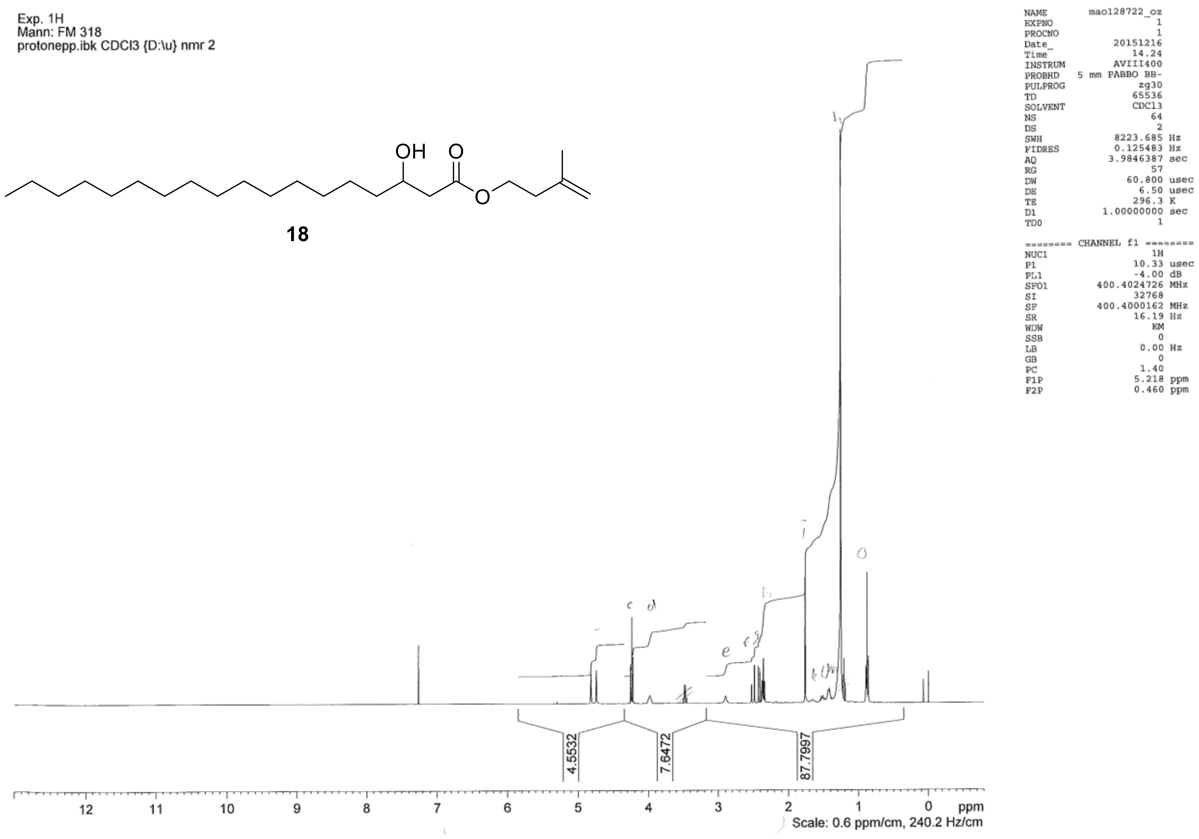

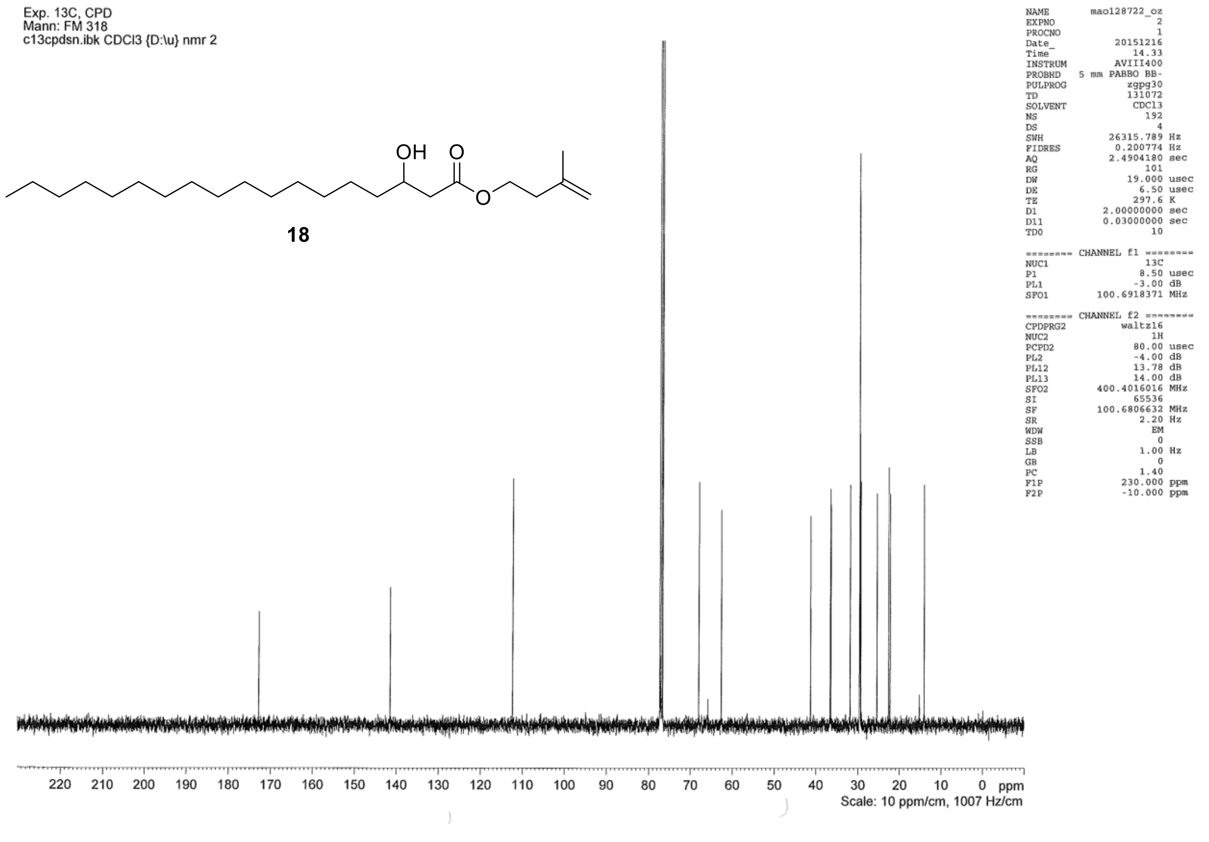

Figure S12. ^{1}H- and ^{13}C-NMR spectra of isoprenyl 3-hydroxyoctadecanoate (18).

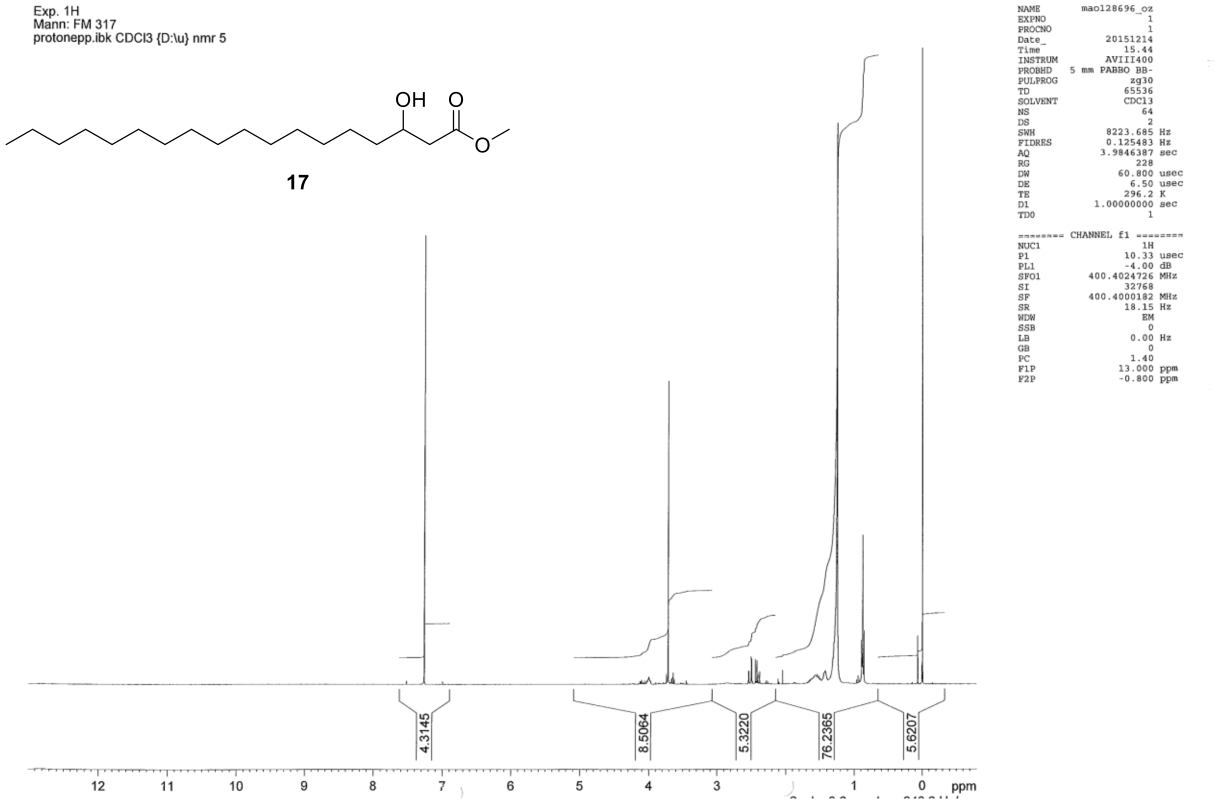

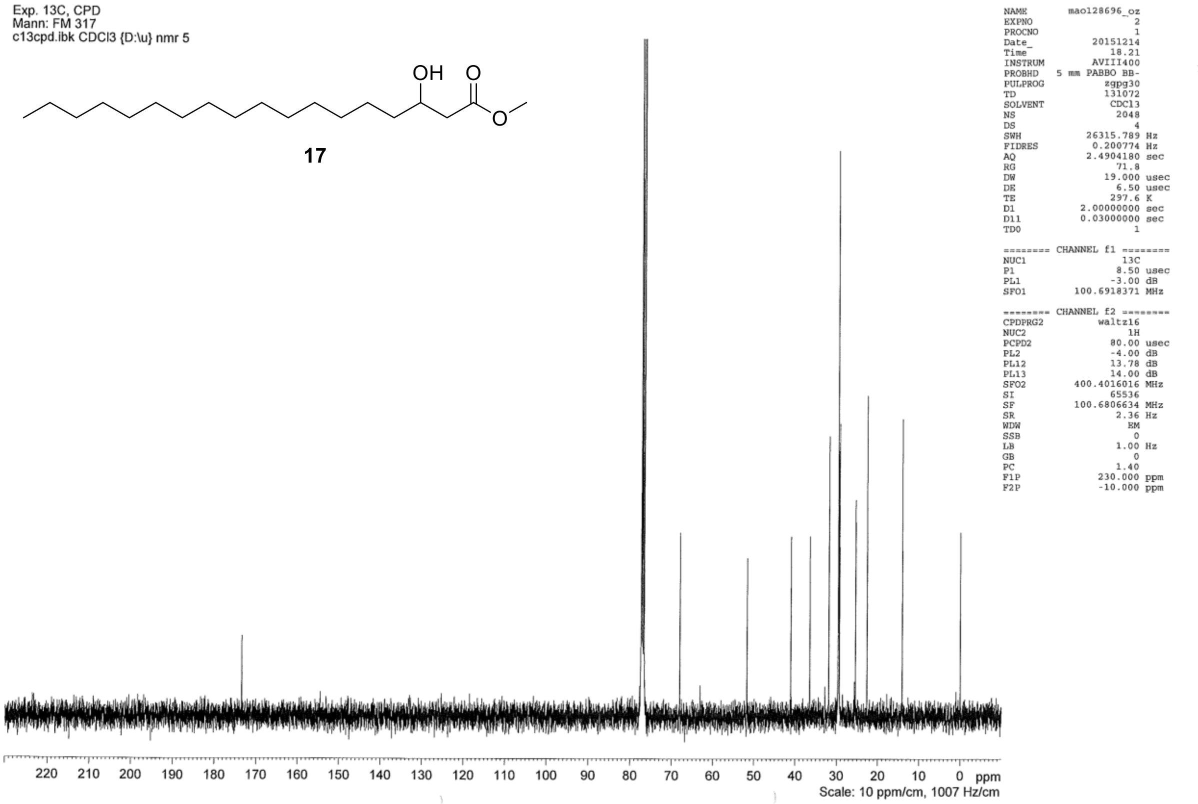

Figure 13. ^{1}H- and ^{13}C-NMR spectra of methyl 3-hydroxyoctadecanoate (17).

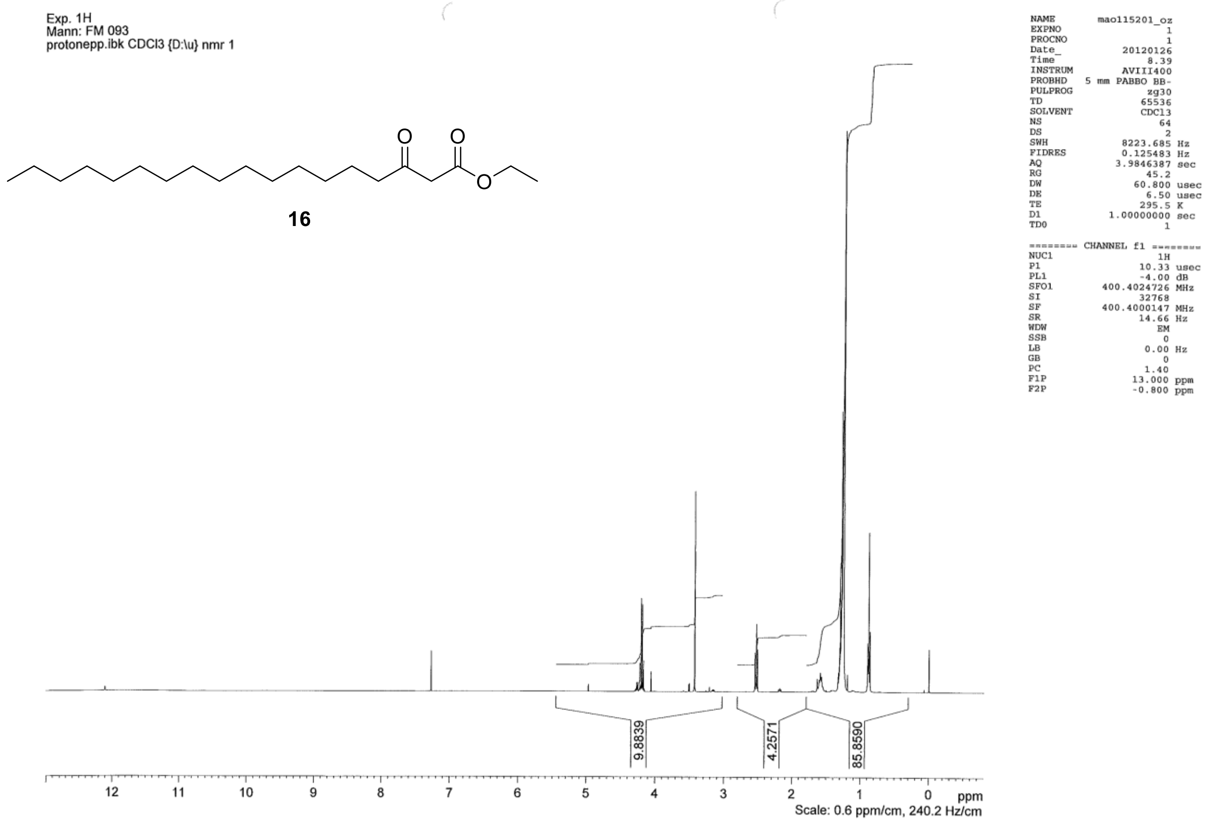

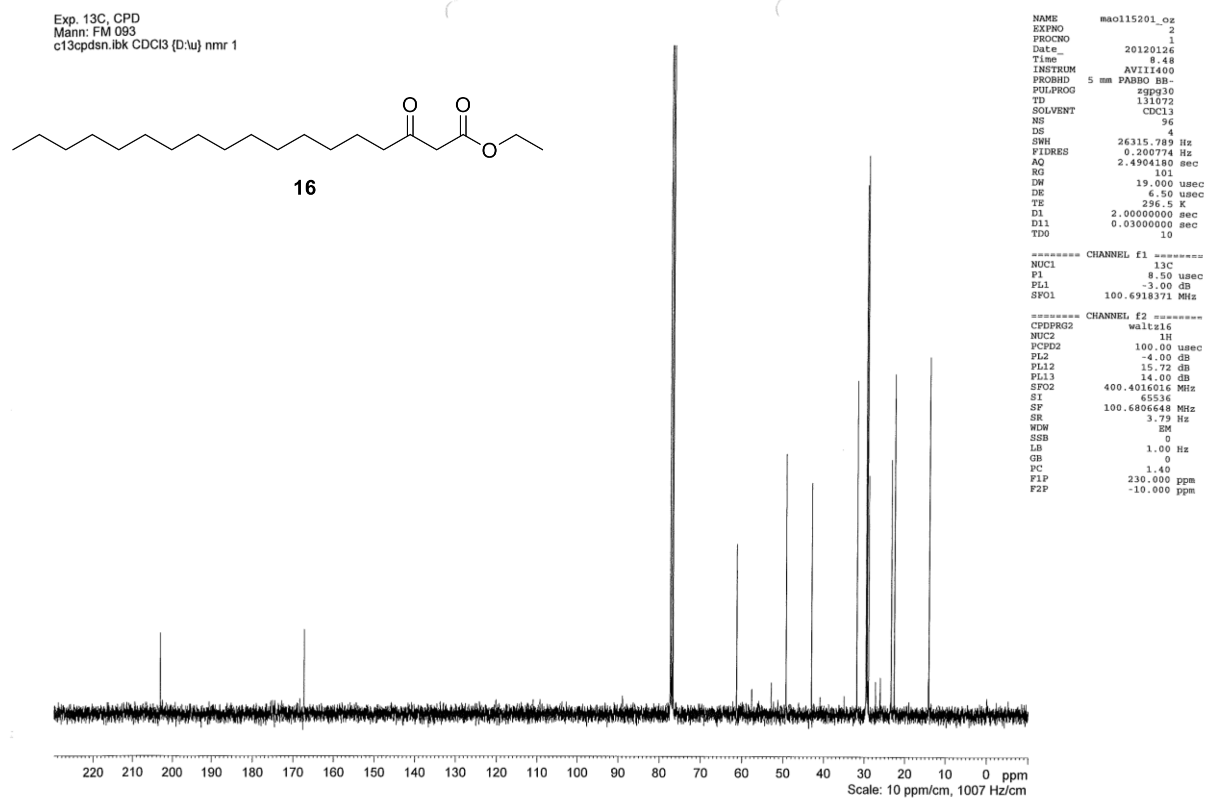

Figure S14. ^{1}H- and ^{13}C-NMR spectra of ethyl 3-oxooctadecanoate (16).

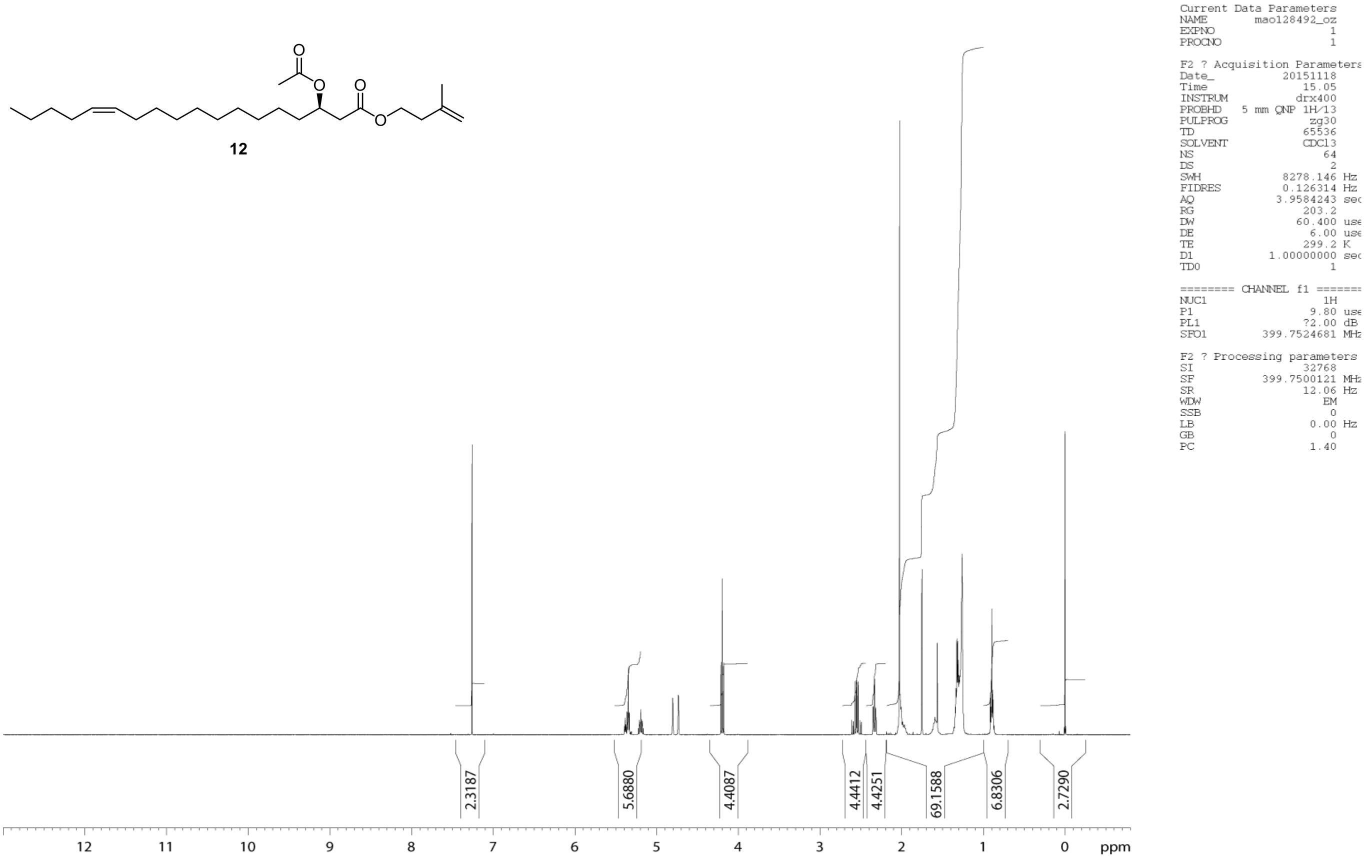

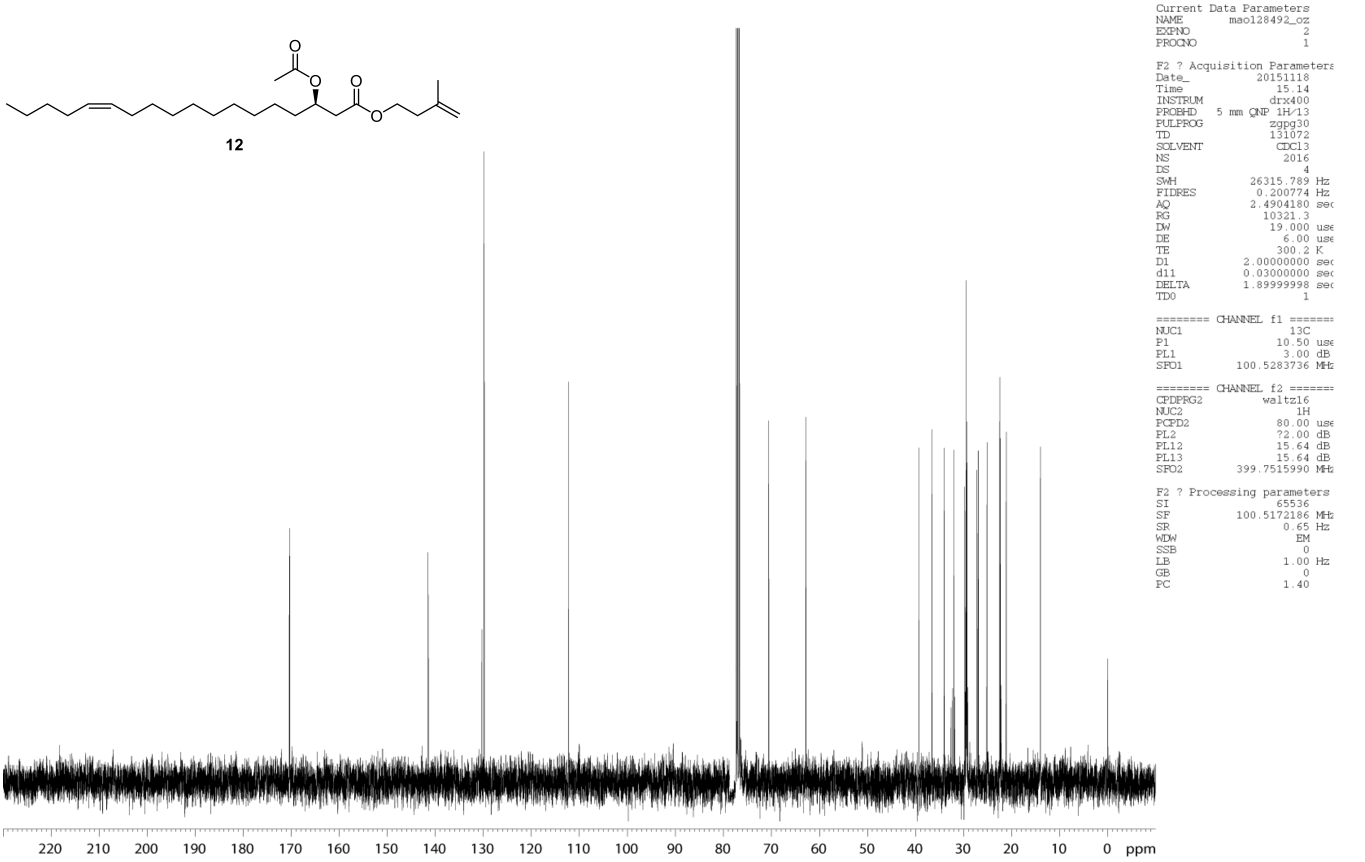

Figure S15. ^{1}H- and ^{13}C-NMR spectra of isoprenyl (3*R*,11*Z*)-3-acetoxy-13-octadecenoate (12).

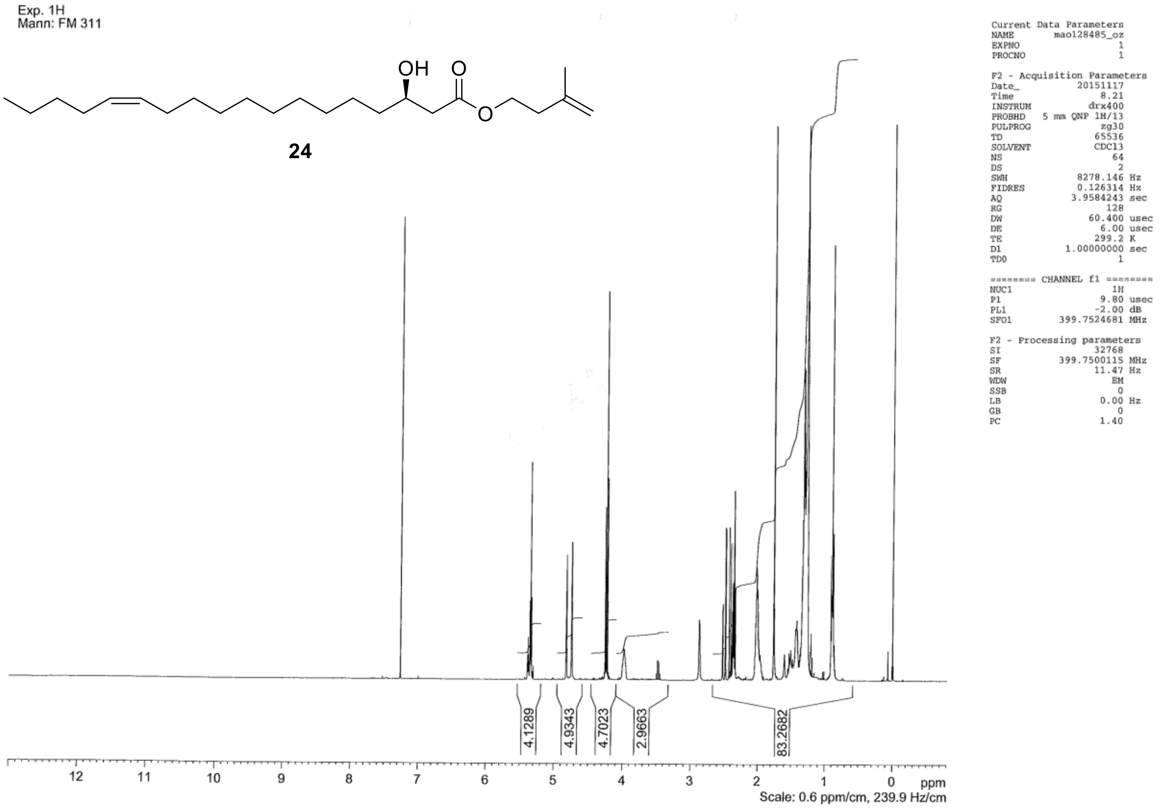

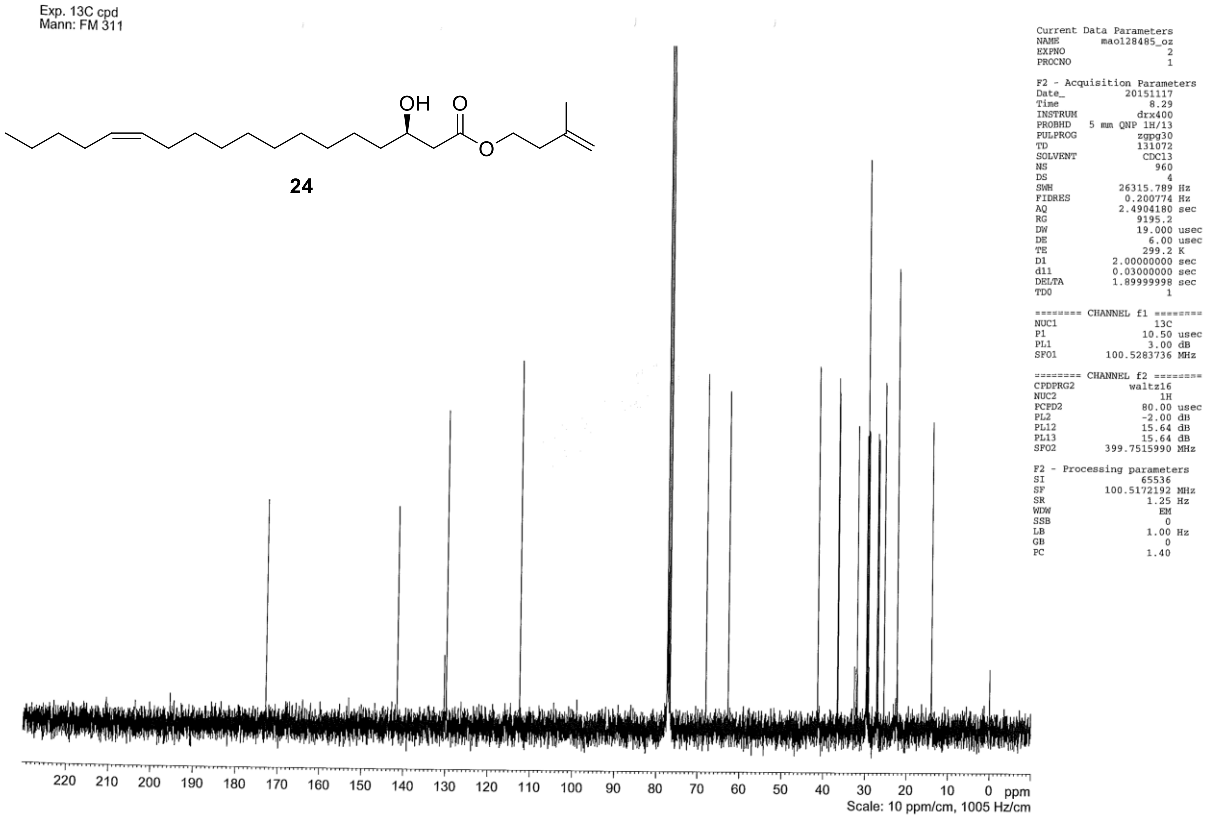

Figure S16. ^{1}**H- and** ^{13}**C-NMR spectra of isoprenyl (3*R*,11*Z*)-3-hydroxy-13-octadecenoate (24).**

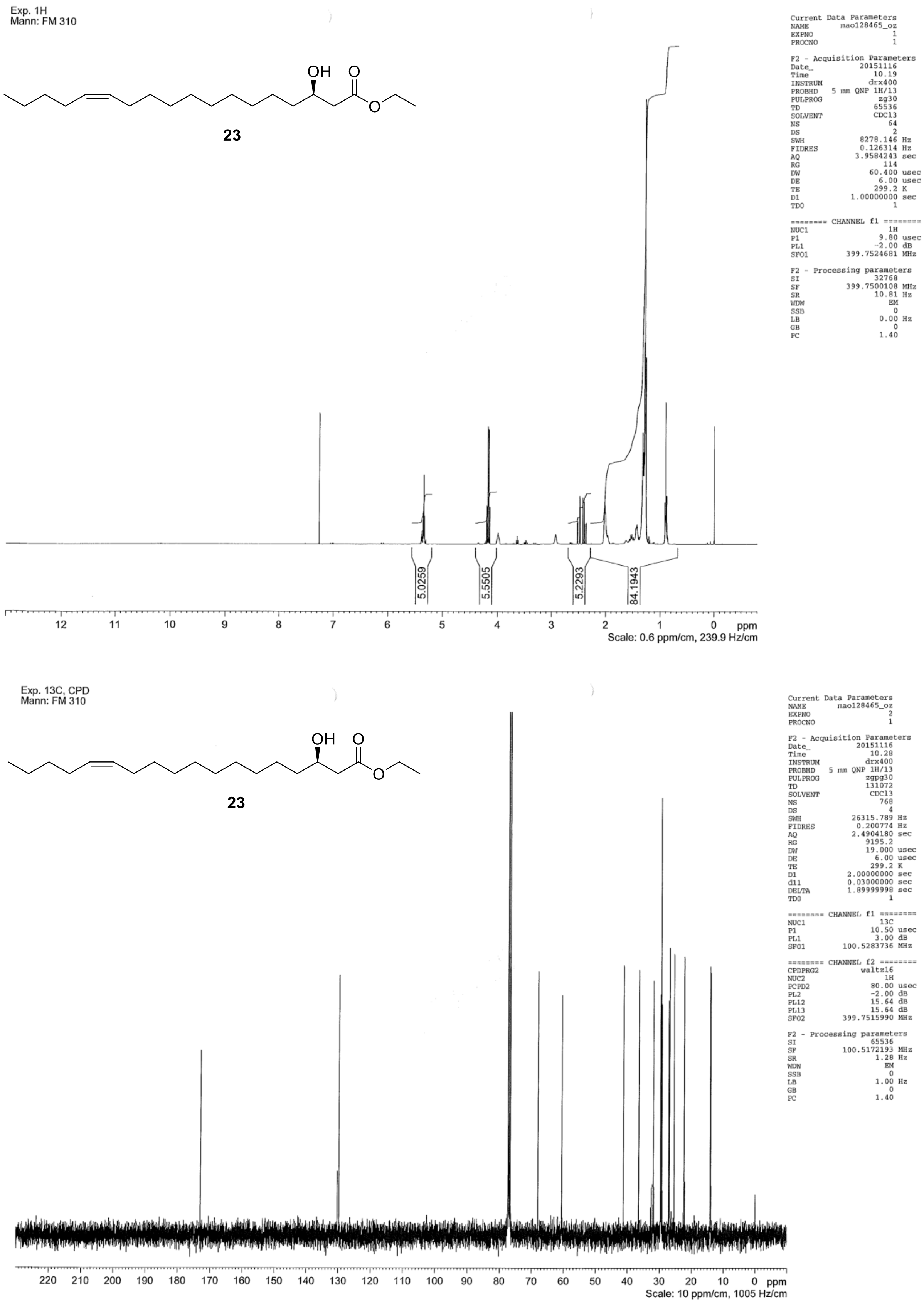

Figure S17. ^{1}H- and ^{13}C-NMR spectra of ethyl (3*R*,11*Z*)-3-hydroxy-13-octadecenoate (23).

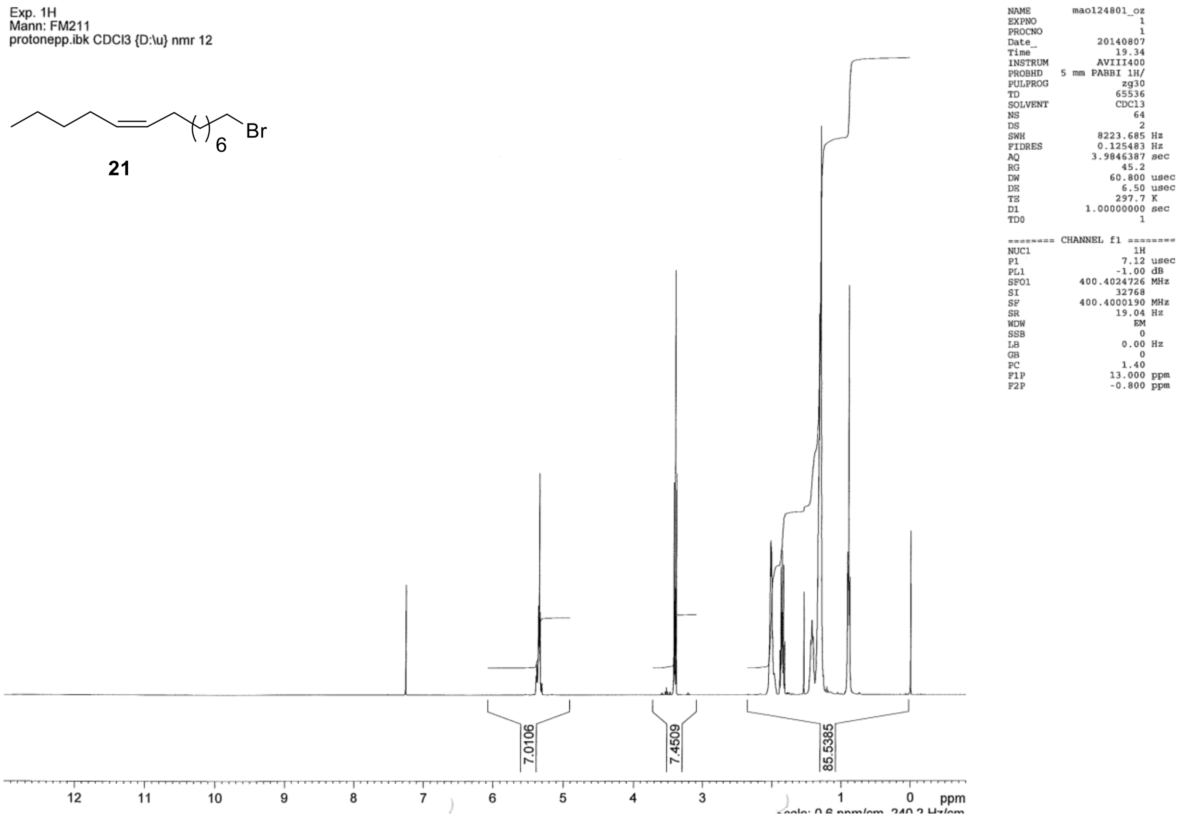

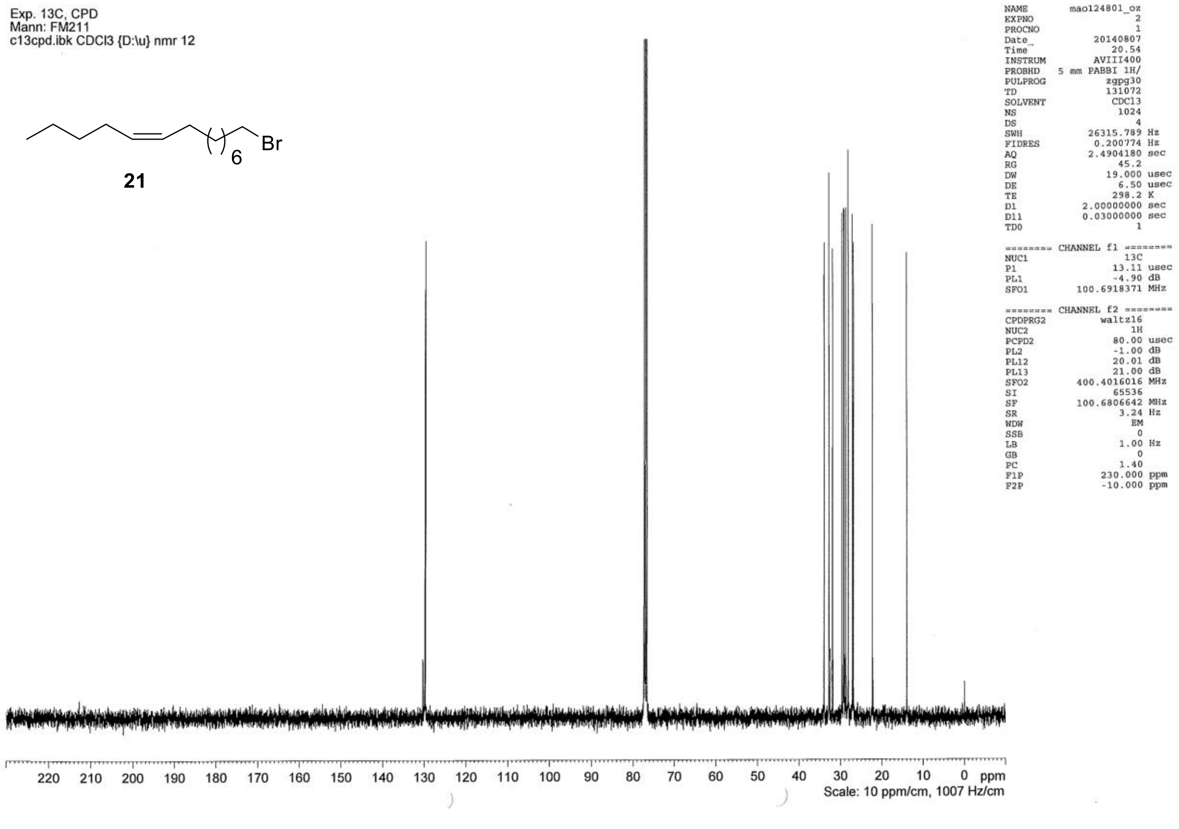

Figure S18. **^{1}H- and ^{13}C-NMR spectra of (*Z*)-14-bromo-5-tetradecene (21).**

6. References

S1. Gauthier, J.; Silva, D. L. de; Gompert, Z.; Whibley, A.; Houssin, C.; Le Poul, Y.; McClure, M.; Lemaitre, C.; Legeai, F.; Mallet, J.; Elias, M. *Mol. Ecol.* **2020,** *29*, 1328–1343.

S2. More, J. D.; Finney, N. S. *Org. Lett.* **2002,** *4*, 3001–3003.

S3. Holmquist, C. R.; Roskamp, E. J. *J. Org. Chem.* **1989,** *54*, 3258–3260.

S4. Otera, J.; Yano, T.; Kawabata, A.; Nozaki, H. *Tetrahedron Lett.* **1986,** *27*, 2383–2386.

S5. Becker, H. G. O.; Beckert, R. *Organikum*. *Organisch-chemisches Grundpraktikum*, 22 edn.; Wiley-VCH: Weinheim, 2004.

S6. Chong, J. W.; Heuft, M. A.; Rabbat, P. *J. Org. Chem.* **2000,** *65*, 5837–5838.

S7. Bestmann, H. J.; Stransky, W.; Vostrowsky, O. *Chem. Ber.* **1976,** *109*, 1694–1700.

S8. Huang, G.; Hollingsworth, R. I. *Tetrahedron: Asymmetry* **1998,** *9*, 4113–4115.
